# EGF Induces Migration Independent of EMT or Invasion in A549 Lung Adenocarcinoma Cells

**DOI:** 10.3389/fcell.2021.634371

**Published:** 2021-03-12

**Authors:** Karin Schelch, Lisa Vogel, Anja Schneller, Jelena Brankovic, Thomas Mohr, Rupert L. Mayer, Astrid Slany, Christopher Gerner, Michael Grusch

**Affiliations:** ^1^Institute of Cancer Research, Department of Medicine I, Medical University of Vienna, Vienna, Austria; ^2^Department of Analytical Chemistry, Faculty of Chemistry, University of Vienna, Vienna, Austria

**Keywords:** cell migration, EGF, TGFβ, lung cancer, epithelial to mesenchymal transition

## Abstract

Tumors and the tumor microenvironment produce multiple growth factors that influence cancer cell behavior via various signal transduction pathways. Growth factors, like transforming growth factor β (TGFβ) and epidermal growth factor (EGF), have been shown to induce proliferation, migration, and invasion in different cell models. Both factors are frequently overexpressed in cancer and will often act in combination. Although both factors are being used as rational targets in clinical oncology, the similarities and differences of their contributions to cancer cell migration and invasion are not fully understood. Here we compared the impact of treating A549 lung adenocarcinoma cells with TGFβ, EGF, and both in combination by applying videomicroscopy, functional assays, immunoblotting, real-time PCR, and proteomics. Treatment with both factors stimulated A549 migration to a similar extent, but with different kinetics. The combination had an additive effect. EGF-induced migration depended on activation of the mitogen-activated protein kinase (MAPK) pathway. However, this pathway was dispensable for TGFβ-induced migration, despite a strong activation of this pathway by TGFβ. Proteome analysis (data are available via ProteomeXchange with identifier PXD023024) revealed an overlap in expression patterns of migration-related proteins and associated gene ontology (GO) terms by TGFβ and EGF. Further, only TGFβ induced the expression of epithelial to mesenchymal transition (EMT)-related proteins like matrix metalloproteinase 2 (MMP2). EGF, in contrast, made no major contribution to EMT marker expression on either the protein or the transcript level. In line with these expression patterns, TGFβ treatment significantly increased the invasive capacity of A549 cells, while EGF treatment did not. Moreover, the addition of EGF failed to enhance TGFβ-induced invasion. Overall, these data suggest that TGFβ and EGF can partly compensate for each other for stimulation of cell migration, but abrogation of TGFβ signaling may be more suitable to suppress cell invasion.

## Introduction

Cell migration is an indispensable function for many cells in multicellular organisms during embryonic development. Controlled cell migration is also critical for processes like wound healing and inflammation throughout adult life. Deregulated cell migration, however, especially in conjunction with the ability to degrade extracellular matrix and invade surrounding tissues, is a hallmark of malignancy and forms the basis for cancer metastasis (Friedl and Wolf, 2003; Chiang and Massague, 2008).

Multiple extracellular stimuli that induce cell migration in diverse cellular contexts have been described (Friedl and Wolf, 2003). Nevertheless, a lot still needs to be discovered about pathway-specific mechanisms and their contribution to increased cell migration. Especially in cancer cells, which often receive a number of potentially pro-migratory signals simultaneously, a better understanding of pathway-dependencies of migration and invasion could potentially lead to more effective antimetastatic therapies. Since the great majority of cancer deaths result from metastasis, this could have important clinical implications (Gandalovicova et al., 2017). We have chosen the KRAS mutated A549 lung adenocarcinoma cell line for investigating how two growth factors, transforming growth factor β (TGFβ) and epidermal growth factor (EGF), which each play fundamental roles in tumor development but activate clearly distinct signaling pathways (Yarden and Sliwkowski, 2001; Schmierer and Hill, 2007), stimulate migration and invasion individually and when acting together. Both growth factors and their receptors are expressed by many cancer cells as well as cells of the tumor microenvironment, including cancer-associated fibroblasts, endothelial cells, and immune cells (Zhang et al., 2014; Zhang S. et al., 2017; Verrecchia and Redini, 2018; Zeng et al., 2019; Garvey et al., 2020). Thus, lung cancer cells and cancer cells arising in other organs have a high likelihood of simultaneously receiving both TGFβ and EGF signals in an autocrine and paracrine manner. The EGF signaling axis is critically involved in tumor cell growth in lung cancer and several other cancer types, e.g., prostate cancer (Liu et al., 2017; Cheaito et al., 2020). The EGF-receptor (EGFR) has become an important target in clinical oncology, and EGFR-targeting kinase inhibitors and monoclonal antibodies are routinely used in the treatment of lung cancer, head and neck cancer, and colorectal cancer (Guardiola et al., 2019). Pharmacological agents blocking TGFβ signaling are being investigated in clinical trials in pancreatic cancer, lung cancer, and hepatocellular carcinoma (de Gramont et al., 2017). Both growth factors have previously been linked to epithelial to mesenchymal transition (EMT), a process by which epithelial cells lose their cell–cell contacts and acquire a fibroblast-like highly motile and invasive phenotype (Kalluri and Weinberg, 2009). EMT has been described as an important contributing process to cancer metastasis (Heerboth et al., 2015). Moreover, EMT was shown to correlate with disease progression and worse prognosis in various cancer types including lung adenocarcinoma (Schliekelman et al., 2015; Cheaito et al., 2019). The interaction of growth factors in EMT and their individual contributions to the process are still not well understood. Filling this knowledge gap could provide additional clues for targeting metastasis.

The results of our study show that TGFβ and EGF have a similar migration-stimulating potential in A549 cells, despite inducing clearly distinct alterations in signal pathway activation, cellular morphology, and protein expression. The combined stimulation with both factors resulted in an additive effect with respect to migrated distance, while selective inhibition experiments showed that both factors can stimulate migration independent of one another. Notably, only TGFβ, but not EGF increased invasion and exhibited protein and transcript expression changes associated with EMT. These results show that stimulation of migration by EGF can occur in an EMT-independent context and suggest that, at least in some cancer cells, the blockade of several independent signals may be required to inhibit migration.

## Materials and Methods

### Cell Culture

A549 lung adenocarcinoma cells were obtained from the American Type Culture Collection (ATCC) and used only for a limited number of passages to ensure cell line identity. Cells were cultivated in RPMI medium containing 10% heat-inactivated fetal bovine serum (FBS) in a humidified atmosphere (37°C, 5% CO_2_). Unless stated otherwise, cells were seeded in medium with 10% FBS and all treatments with cytokines and inhibitors were started 24 h later in medium without FBS.

### Cytokines and Drugs

Epidermal growth factor and TGFβ were purchased from Sigma and Peprotech and used at concentrations of 50 and 5 ng/mL, respectively, following previous literature reports (Kasai et al., 2005; Buonato et al., 2015). For treatments combining growth factors with specific signaling pathway inhibitors, the following compounds were added 1 h before cytokines at the indicated final concentrations: the TGFβ receptor type I inhibitor SB-431542 (Tocris, 20 μM), the EGFR inhibitor erlotinib (Selleckchem, 10 μM), the PI3K inhibitor LY-294002 (Selleckchem, 20 μM), the AKT inhibitor MK-2206 (Selleckchem, 5 μM), the MEK inhibitor U0126 (Selleckchem 10 μM), the FAK inhibitor BI-853520 (Boehringer Ingelheim, 5 μM), and the ROCK inhibitor Y-27632 (Selleckchem, 10 μM). Concentrations were chosen according to literature reports and produced only mild cytotoxicity over the treatment period. PBS and DMSO were used as vehicle controls for growth factors and inhibitors, respectively.

### Videomicroscopy

Videos were generated using a Nikon Visitron Live Cell System (Visitron Systems GmbH) with images taken every 5 min for 72 h. Migration and cell cycle of single cells were manually tracked using ImageJ to obtain coordinates for each individual cell and time point. For further analysis of migratory behavior including speed, mean squared displacement (MSD), directionality ratio (DR), and origin plots, the DiPer migration tool for Microsoft Excel was used. The algorithms used by this program have been described in detail by Gorelik and Gautreau (2014).

### Morphology Analysis

Cells (1 × 10^5^) were seeded into six-well plates and treated the next day as indicated. Microscopic images were taken 48 h later using a Nikon Eclipse Ti300 microscope and Digital Sight camera. Alternatively, respective images obtained from videomicroscopy were used. Cell outlines were manually traced in ImageJ for calculation of shape descriptors. Aspect ratio (major axis/minor axis), circularity [4π(area/perimeter)^2^], area and maximum diameter of single cells, and the distance to the nearest neighbor were then determined using ImageJ.

### Immunofluorescence

Cells (1.5 × 10^4^) were seeded into eight-well chamber slides and treated on the next day as indicated with TGFβ, EGF, or a combination of both. Forty-eight hours later, cells were fixed with 4% PFA for 15 min, blocked for 1 h (5% BSA, 0.3% Triton X in PBS), and then incubated with a monoclonal anti-tubulin antibody (Sigma, T5168, 1:2000 in 1% BSA, 0.3% Triton X in PBS) for 1 h at RT. This was followed by anti-mouse-FITC (1:100, Sigma, F5262) and phalloidin-TRITC (1:1000, Sigma, P1951) treatment for 1 h at RT. Cells were embedded in Vectashield mounting medium containing DAPI (Vector laboratories), and slides were imaged with a Zeiss LSM 700 confocal microscope using a 63x oil immersion lens.

### Sprouting Assay

Cells (5 × 10^3^) were seeded into 96-well U-bottom suspension plates in medium containing 20% methyl cellulose solution (1.2% in serum-free medium) and allowed to form spheroids for 48 h. Meanwhile, a 96-well plate was coated with 50 μL 1% agarose. Spheroids were then mixed with collagen resulting in 100 μL medium containing 10% methylcellulose and 1 mg/mL collagen, which was placed on top of the agarose. After solidification of the mixture, another 100 μL medium containing the treatment (2.5x) was added on top, resulting in a total volume of 250 μL per well. Pictures were taken after 0, 24, 48, 120, and 144 h on a Nikon Eclipse Ti300 microscope, and the mean sprout length of 10 representative sprouts, each from at least nine spheroids, was measured using ImageJ.

### Proliferation Assay

Cells (3 × 10^3^) were seeded into 96-well plates. On the next day, EGF, TGFβ, or both were added as indicated. Plates were frozen at -80°C after 72 h. For quantification, an SYBR green-based assay was used as previously published (Schelch et al., 2018a). Briefly, plates were thawed, lysis buffer (10 mM Tris/HCl pH = 8, 2.5 mM EDTA, 0.1% Triton X-100) containing SYBR green (10,000x, Thermo Fisher Scientific, 1:8000) was added, and after incubation for 2 h at RT, fluorescence was read on an Infinite M200 Pro Photometer (Tecan).

### PCR Analysis

RNA was isolated using the InnuPrep RNA Kit (Analytik Jena) 24 h after treatment with TGFβ, EGF, or a combination of both and reverse transcribed with M-MLV reverse transcriptase (Thermo Fisher Scientific). SYBR green-based qPCR was performed on a CFX96 Thermocycler (BioRad) using iTaq universal SYBR green super mix (BioRad). Primer pairs are listed in Supplementary Table S1. Changes in gene expression are shown as log2 of 2^–ΔΔ*Ct*^ compared to the respective untreated control and normalized to GAPDH which was used as reference gene.

### Immunoblots

Cells (1 × 10^5^) were seeded into six-well plates and incubated with EGF, TGFβ, or both. Cells were harvested in lysis buffer (150 mM NaCl, 50 mM HEPES, 10% glycerol, 1 mM EDTA, 0.5 mM Na_3_VO_4_, 10 mM NaF, 1% Triton X-100, and 1.5 mM MgCl_2_); proteins were separated by SDS-PAGE and electroblotted onto PVDF membranes. Immunoblots were performed as described (Schelch et al., 2014). In brief, membranes were blocked for 1 h at ambient temperature with 5% skimmed milk or BSA following the recommendations of the respective primary antibody manufacturers and subsequently incubated overnight at 4°C with primary antibodies. On the next day, membranes were washed with TBST and incubated with HRP-coupled secondary antibodies for 1 h at room temperature. Blot development was done with Biorad Clarity Western ECL Substrate, and luminescent signals were recorded on X-ray film. The following primary antibodies were used 1:1000: pAkt (Cell Signaling, #4060), Akt (Cell Signaling, #4691), pErk (Cell Signaling, #9101), Erk1/2 (Cell Signaling, #4695), pSmad2 (Cell Signaling, #31085), Smad2 (Cell Signaling, #5339), and β-Actin (Sigma, A5541). Band intensity quantification was done with ImageJ.

### Proteome Analyses

Cells (1 × 10^6^) were seeded into 10 cm petri dishes. Treatment with TGFβ, EGF, or both started 24 h later and lasted for 48 h. Cell morphology was microscopically checked before harvesting the cells. Cell supernatants were collected and proteins precipitated overnight with ethanol at −20°C. The cells remaining in the dishes were further processed in order to obtain cytoplasmic and nuclear proteins, as previously described (Slany et al., 2014). In short, cells were lyzed in isotonic lysis buffer supplemented with protease inhibitors and mechanical shear stress was applied by pressing the cells through a 23 g syringe. Cytoplasmic and nuclear proteins were extracted separately and precipitated overnight with ethanol at −20°C. After centrifugation, all protein samples were dissolved in sample buffer (7.5 M urea, 1.5 M thiourea, 4% CHAPS, 0.05% SDS, and 100 mM dithiothreitol). Protein concentrations were determined via Bradford assays (Bio-Rad Laboratories), and in-solution digestion of proteins was performed with trypsin (Roche Diagnostics) (Muqaku et al., 2017).

Digested peptides were analyzed as previously described (Muqaku et al., 2017). In short, 1 μL of peptide solution was loaded and peptides were separated by liquid chromatography on an UltiMate 3000 RSLC nano System (Dionex). Data acquisition was conducted on a QExactive mass spectrometer (Thermo Fischer Scientific) using a top-8 data-dependent method described previously (Slany et al., 2016). Protein identification was achieved using the MaxQuant 1.5.2.8 software (Cox and Mann, 2008) employing the Andromeda search engine (Cox et al., 2011) and searching against the UniProt database for human proteins (version 102014 with 20,195 entries). Including data obtained from three biological replicates (independent cell experiments) as well as technical replicates (independent injections into LC-MS system), an MS1-based label-free quantification (LFQ) approach and statistical analysis were applied to quantify the identified proteins based on LFQ values using Perseus (Cox and Mann, 2012). After the removal of potential contaminants, reversed sequences, and proteins only identified by site, the logarithmic LFQ values on the base 2 were calculated and averaged for technical replicates. Invalid values were filtered using the criteria of valid values in at least 90% of samples per treatment group in at least one group. Missing values were replaced from a normal distribution, and *t*-tests were performed (FDR < 0.05, S0 = 0.5). Identified proteins are listed in Supplementary Table S2. Venn diagram analysis was performed with Venny 2.1 (Oliveros, J.C. 2007-2015 Venny. An interactive tool for comparing lists with Venn’s diagrams^1^.). Unsupervised clustering was performed with Morpheus^2^. Pathway analysis was performed with the DAVID 6.8 functional annotation tool (Huang da et al., 2009a, b). Heatmaps were generated with Prism 8.0 (GraphPad), and spider plots were created with MS Excel. Lists of proteins included in specific biological process categories related to cell motility were downloaded from the MSig Database^3^.

### Statistical Analysis

Data on cell behavior are from at least three independent experiments each and were analyzed for statistical differences by one-way ANOVA with Sidak’s or Dunnett’s multiple comparisons test with Prism 8.0 (GraphPad). A *P*-value < 0.05 was considered significant.

## Results

### TGFβ and EGF Stimulate Cell Migration Alone and in Combination

**FIGURE 1:**
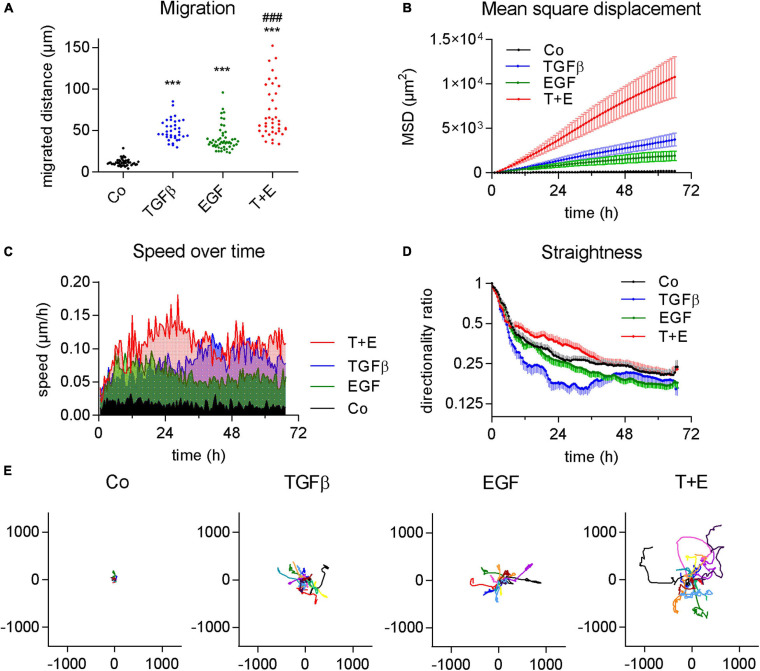
TGFβ and EGF stimulate cell migration alone and in combination. Cells were treated with TGFβ (5 ng/mL), EGF (50 ng/mL) or a combination of both (T + E), and live cell videomicroscopy was performed over 72 h with pictures taken every 5 min. This experiment was performed at least five times with two to four technical replicates. **(A)** Dots represent cumulative migrated distance of individual cells (36 < *n* < 51) over 72 h, assessed by manual single cell tracking using Image J. ***P < 0.001 treatment versus control, ^###^*P* < 0.001 T + E versus EGF, and T + E versus TGFβ, One-way ANOVA with Sidak’s multiple comparisons test. **(B)** Mean squared displacement (MSD), **(C)** average speed, **(D)** directionality ratio, and **(E)** origin plots of representative tracked cells were calculated by DiPer. Lines represent means and SD in **(B,D)**, means only in **(C)**, and individual cells in **(E)**.

To compare the impact of two important growth factors on cancer cell migration, we treated A549 cells that were kept in serum-free growth medium with either TGFβ, EGF, or a combination of both. All three treatments led to a significant increase in migrated distance over a course of 3 days compared to vehicle-treated control A549 cells, as shown by videomicroscopy analysis. All cells of the treated cell populations showed increases in migration versus the control (Figure 1A). The extent of this increase was similar in both TGFβ- and in EGF-treated cultures, while the combination treatment showed a further increase of migration distance (Figure 1A). Differences were even more pronounced in the MSD, describing the mean squared area that a cell covers over time (Figure 1B).

Although the increases in migrated distance were comparable between TGFβ and EGF (Figure 1A), we observed notable differences when we analyzed other aspects of migratory behavior. We found that EGF-treated cells reached their maximum speed at about 6 h which slightly dropped again at around 20 h after treatment (Figure 1C). TGFβ, in contrast, increased cell speed at a slower rate and plateaued at around 36 h. Treatment with both growth factors together resulted in additive effects with a much higher speed than either of the single treatments from around 12–36 h (Figure 1C). This suggests that the induction of migration by EGF happens via a different, faster pathway than induction by TGFβ. We also assessed the straightness of cell migration by calculating the DR over time (Figure 1D). While a value of 1 represents a totally straight path, lower values describe progressively less straight migration. TGFβ resulted in a distinctly lower DR than EGF treatment. The combination treatment showed the highest DR values (Figure 1D). The migrated distance, as well as both the MSD and DR, are well reflected by origin plots which show 10–15 representative tracks of single cells relative to one origin (0/0) (Figure 1E).

### Increased Migration in Response to TGFβ and EGF Is Linked to Clearly Distinct Alterations in Cell Morphology

**FIGURE 2:**
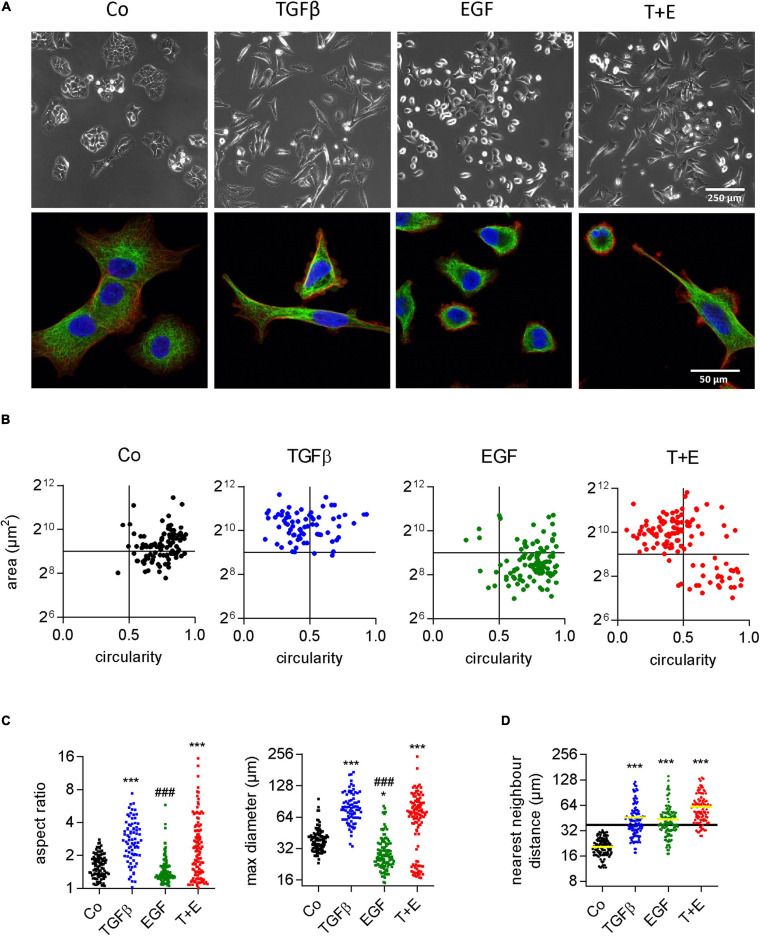
Increased migration in response to TGFβ and EGF is linked to distinct alterations in cell morphology. **(A)** Representative phase contrast (upper panels) and confocal (lower panels, blue: DAPI, red: actin, green: tubulin) microscopic images of A549 cells 48 h after the indicated treatments [TGFβ (5 ng/mL), EGF (50 ng/mL), or a combination of both (T + E)]. Shape descriptors of single cells (77 < *n* < 115) including **(B)** area versus circularity, **(C)** aspect ratio and max. diameter, and **(D)** distance to nearest neighbor were determined using Image J. Dots represent individual cells. ***P < 0.001, *P < 0.05 treatment versus control, ^###^*P* < 0.001 EGF versus TGFβ, One-way ANOVA with Sidak’s multiple comparisons test.

Although both growth factors stimulated cell migration to a similar extent, they produced remarkable differences with respect to cell morphology changes (Supplementary Video S1). TGFβ changed the shape of A549 cells from a cubic into a more elongated, angular form with many protrusions. EGF treatment, in contrast, resulted in round, sphere-shaped cells reminiscent of dividing cells. When TGFβ and EGF were combined, both cell shapes could be observed, as shown by representative phase contrast and high-resolution confocal microscopic images (Figure 2A and Supplementary Figures S1A–D). With respect to migration, the fastest moving cells in the combination group were those with the EGF-induced shape. We used circularity and area of single cells as additional shape descriptors to better visualize the morphological alterations following growth factor treatments (Figure 2B). TGFβ-treated cells were bigger and more elongated than the vehicle-treated cells, whereas EGF treatment resulted in smaller and less elongated cells. The combination of TGFβ and EGF showed a mix of both populations. Similar results were obtained when aspect ratio and maximum diameter were assessed for all treatments (Figure 2C). Cell scattering was then assessed by nearest neighbor distance. In contrast to cell shape changes, cell scattering was induced by TGFβ and EGF to the same extent (Figure 2D).

### Prolonged Phosphorylation of Erk Is Induced by TGFβ but Not EGF

**FIGURE 3:**
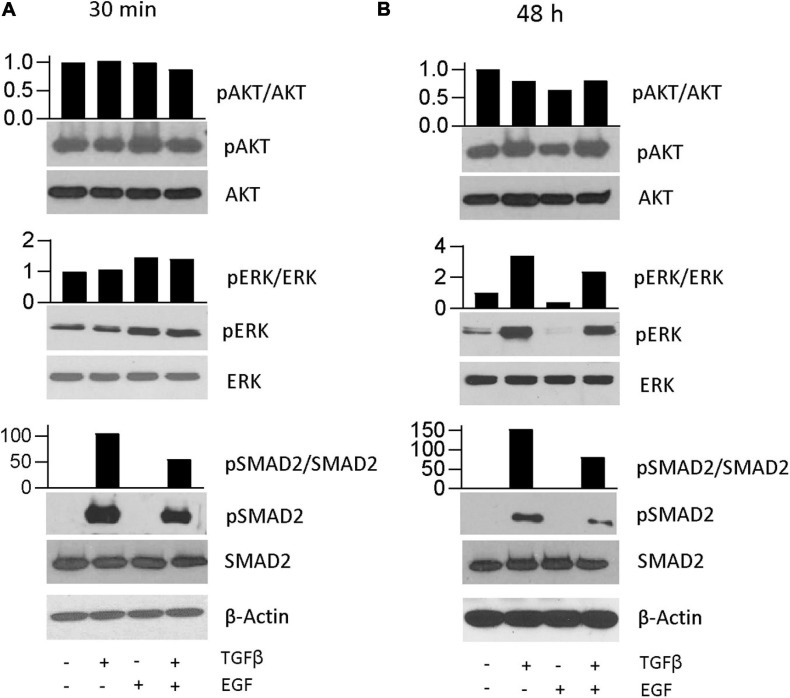
Prolonged phosphorylation of Erk is induced by TGFβ but not EGF. Representative immunoblots of A549 cells **(A)** 30 min or **(B)** 48 h after treatment with TGFβ, EGF, or a combination of both. One example and means of densitometry data normalized to control from three experiments are shown. Beta actin was used as a control for equal sample loading. Scans of uncropped immunoblots of all three replicates are shown as Supplementary Data.

To analyze the involvement of the respective canonical signal transduction pathways in TGFβ- and EGF-induced effects, we performed immunoblots after short-term (30 min) and prolonged (48 h) treatment. TGFβ led to a strong induction of Smad2 phosphorylation, as expected, but no increase in either pErk or pAkt levels after short-term treatment was seen (Figure 3A). Since A549 cells have a mutation in KRAS, the control had a relatively high basal pErk level. However, when treated with EGF and the combination, a further increase in pErk but not pAkt was seen in the short-term experiment. Basal and EGF treatment-induced Erk phosphorylation depended on MEK activity, since they were blocked by MEK inhibition (Supplementary Figure S2). These phosphorylation patterns were distinctly altered after 48 h of treatment (Figure 3B). While pSmad2 remained elevated in treatment groups that had received TGFβ alone or in combination with EGF, these two groups also had increased levels of pErk but not pAkt. In contrast, neither pErk levels nor pAkt levels were upregulated in the EGF-treated group compared to the control (Figure 3B).

### EGF but Not TGFβ Depends on MAPK Signaling for Stimulation of Migration and Morphological Alterations

**FIGURE 4:**
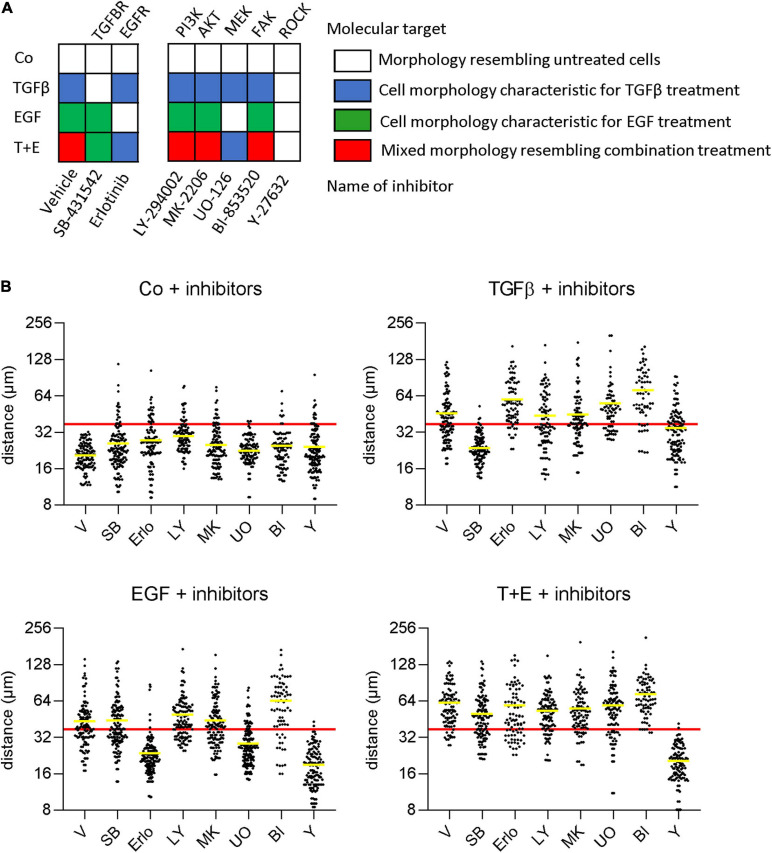
EGF but not TGFβ depends on MAPK signaling for stimulation of migration and morphological alterations. **(A)** Cells were pre-treated with the indicated inhibitors or vehicle 1 h before treatment with TGFβ, EGF, or both (T + E) and images were recorded after 48 h. Growth factor-specific alterations in cell morphologies (as described in Figure 2A) are indicated by the respective colors. **(B)** Cells were treated as above with SB-431542 (SB), erlotinib (Erlo), LY-294002 (LY), MK-2206 (MK), U0126 (U0), BI-853520 (BI), Y-27632 (Y), or vehicle (V) plus the respective growth factors, and distance to nearest neighbors was assessed using Image J. Data are shown as scatter plots and mean (yellow line) of >100 individual cells. The mean diameter of a non-treated cell (37 μm) is shown as a red horizontal line and was chosen as cut-off for the mean to describe cell scattering (Bakiri et al., 2015; Selvaraj et al., 2015; Hyakusoku et al., 2016).

To determine which specific signaling cascades are required for the observed growth factor-induced changes in cell morphology and scattering, we pre-treated cells with small-molecule inhibitors of downstream signaling proteins before growth factor treatment. In addition to the changes in cell morphology (Figure 4A), we determined cell scattering using the nearest neighbor distance (Figure 4B). As expected, inhibition of the TGFβ receptor and the EGFR prevented the effects of TGFβ and EGF, respectively. The combination group displayed effects specific for the respective not-inhibited growth factor. From the panel of downstream inhibitors, inhibition of MEK, but not the PI3K/Akt pathway could prevent the changes induced by EGF. This suggests that a functioning mitogen-activated protein kinase (MAPK) pathway is crucial for EGF-induced morphology changes and increased motility. On the other hand, MEK activity was not required for TGFβ-induced morphology changes and scattering, despite the strong upregulation of pErk levels by TGFβ after 48 h. Further, the inhibition of FAK had no effect on morphology or migration changes in either treatment. Inhibition of ROCK, in contrast, impaired both the EGF- and TGFβ-induced effects, suggesting that this protein is an important downstream effector of migration for both growth factors.

### TGFβ and EGF Regulate Partially Overlapping Sets of Migration- and Metastasis-Related Proteins

**FIGURE 5:**
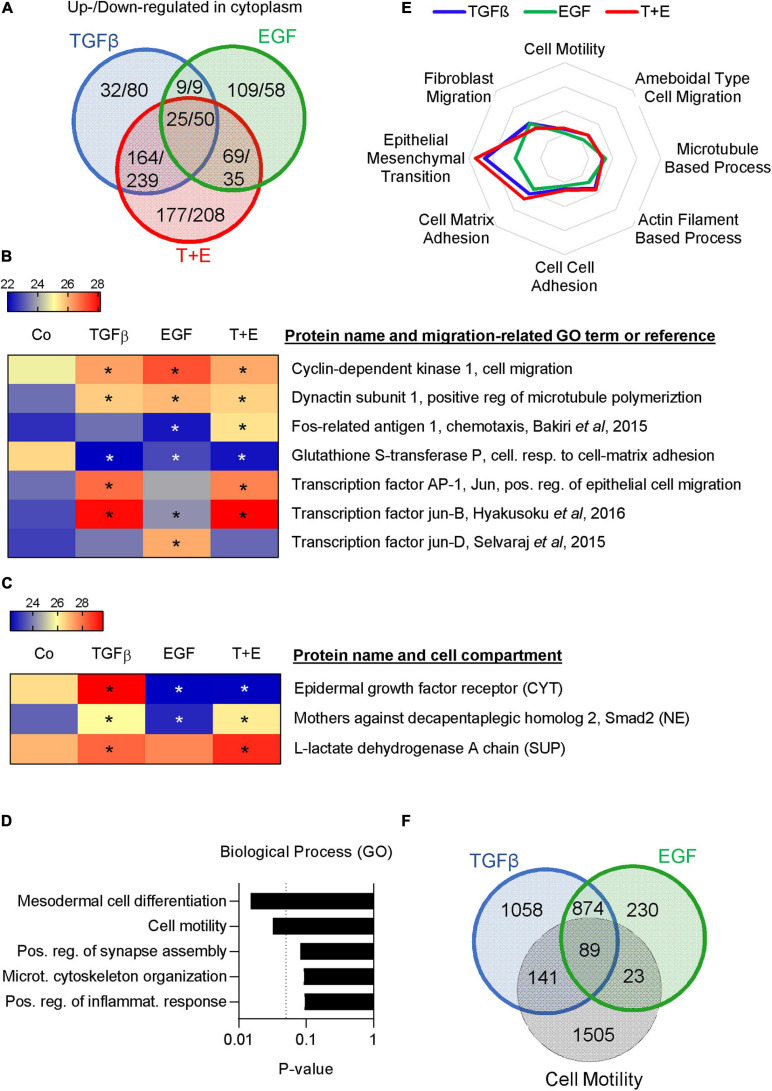
TGFβ and EGF regulate partially overlapping sets of migration- and metastasis-related proteins. Proteomics analysis was performed in triplicates with two technical replicates. **(A)** Venn diagram showing the numbers of upregulated/downregulated proteins (cut-off: q < 0.25) in the cytoplasmic fraction of A549 cells 48 h after treatment with TGFβ, EGF, or a combination of both compared to the vehicle-treated control. **(B)** Heatmap showing expression levels of selected migration-related proteins in the nuclear fraction [LFQ values, * indicates q < 0.25 compared to control (Co)]. **(C)** Heatmap showing the expression of EGFR in the cytoplasm (CYT), Smad2 in the nucleus (NE), and LDH in the supernatant (SUP). **(D)** Upregulated proteins in the cytoplasmic fraction shared by TGFβ, EGF, and the combination were subjected to GO term analysis and the top five biological process categories ranked by *P*-value are shown. **(E)** Up- and downregulated proteins in the cytoplasm, nucleus, and supernatant were pooled for each treatment. Overlaps with proteins in different migration-related GO-term categories were calculated and are shown as spider plot. **(F)** Venn diagram showing the overlap of pooled up- and downregulated proteins in all three cell fractions after TGFβ or EGF treatment and proteins represented in the GO term “cell motility.”

To further compare the underlying mechanisms of TGFβ- and EGF-induced cell migration in A549 cells, we used a proteomics approach. The cytoplasmic fractions, nuclear fractions, and culture supernatants were analyzed 48 h after treatment with TGFβ, EGF, or the combination. Positive hits in each fraction were identified using cut-off parameters of q-value < 0.25 (Supplementary Table S3). In the cytoplasm, 230 proteins were upregulated and 378 downregulated by TGFβ, 212 upregulated and 152 downregulated by EGF, and 435 upregulated and 532 downregulated by the combination (Figure 5A and Supplementary Table S4). The respective numbers of up- and downregulated proteins in the nuclear fractions and in the supernatants are shown in Supplementary Figure S3 and Supplementary Tables S5, S6. Overall, TGFβ changed a higher number of proteins than EGF in all three cellular compartments. Proteins up- or downregulated by one growth factor were more likely to be altered in the same direction by the other growth factor. For example, of the 439 proteins upregulated by EGF in the nuclear fraction, 278 (63%) were also upregulated by TGFβ, whereas only 33 (7.5%) were downregulated by TGFβ. The combination treatment showed a much bigger overlap in altered proteins with TGFβ than with EGF treatment. This was also reflected in an unsupervised clustering analysis performed with the logarithmic LFQ intensities, where TGFβ and the combination clustered together in all cell compartments, whereas EGF was closer to the control (Supplementary Figure S4).

Various cell migration-related proteins in the nuclear fractions (Figure 5B) and the cytoplasmic and supernatant fractions (Supplementary Figure S5) were altered in a treatment-specific way. For example, treatment with TGFβ and the combination stimulated expression of Matrix Metalloproteinase 2 (MMP2), N-cadherin, and the transcription factor Jun, while EGF did not. On the other hand, EGF increased Jun-D and the EGFR ligand Amphiregulin. A number of proteins linked to cell motility like CDK1, LIF, and Dynactin subunit 1 were upregulated by each of the three treatments, while some, like FOSL1, were only altered by the combination. Notably, we observed the expected downregulation of EGFR in the cytoplasm of samples that had received EGF and an increase in nuclear Smad2 in samples treated with TGFβ (Figure 5C). Interestingly, an increase of lactate dehydrogenase (LDHA) was observed in the supernatant of all growth factor-treated samples, which is indicative of increased cell death.

A gene ontology (GO) term analysis performed on commonly upregulated proteins in the cytoplasm across all three treatments showed that “cell motility” and “cytoskeletal organization” were the second and fourth most significantly associated pathways (Figure 5D). Cytoplasmic proteins upregulated only by EGF but not by TGFβ were strongly associated with translation, whereas those regulated by TGFβ but not EGF or exclusively by the combination treatment were linked to cell–cell and cell–matrix adhesion (Supplementary Figure S5). To focus specifically on migration-related proteins, we investigated the overlap of all TGFβ-regulated, all EGF-regulated, and all combination-regulated proteins from Supplementary Table S3 with proteins represented in various migration-related GO terms. The results indicate a marked difference with respect to the GO term EMT, which showed a higher overlap with TGFβ-regulated proteins compared to EGF-regulated proteins, whereas other categories showed little difference (Figure 5E). Of the TGFβ-regulated proteins, 230 were represented in the cell motility category. Of those, 89 were shared with the 112 EGF-regulated proteins represented in this category (Figure 5F). Thus, the motility proteins shared by TGFβ and EGF represent the majority (79%) of the EGF-regulated motility proteins but only 39% of the TGFβ-regulated motility proteins. We then submitted TGFβ-regulated and EGF-regulated motility proteins for GO term analysis. As expected, the top five GO terms associated with TGFβ- and EGF-regulated motility proteins showed a high degree of overlap (Supplementary Figure S7). However, “positive regulation of cell migration” was the top GO term associated with TGFβ-regulated motility proteins and the top GO term for EGF-regulated motility proteins was “leukocyte migration.”

Overall, the proteomics data suggested that (i) TGFβ and EGF regulate distinct but partially overlapping sets of cell adhesion and motility proteins, (ii) all treatments increase cell death to some extent, and (iii) TGFβ has a higher capacity to induce extracellular matrix degradation.

### All Treatments Decrease Cell Number and the Combination Increases Doubling Time

**FIGURE 6:**
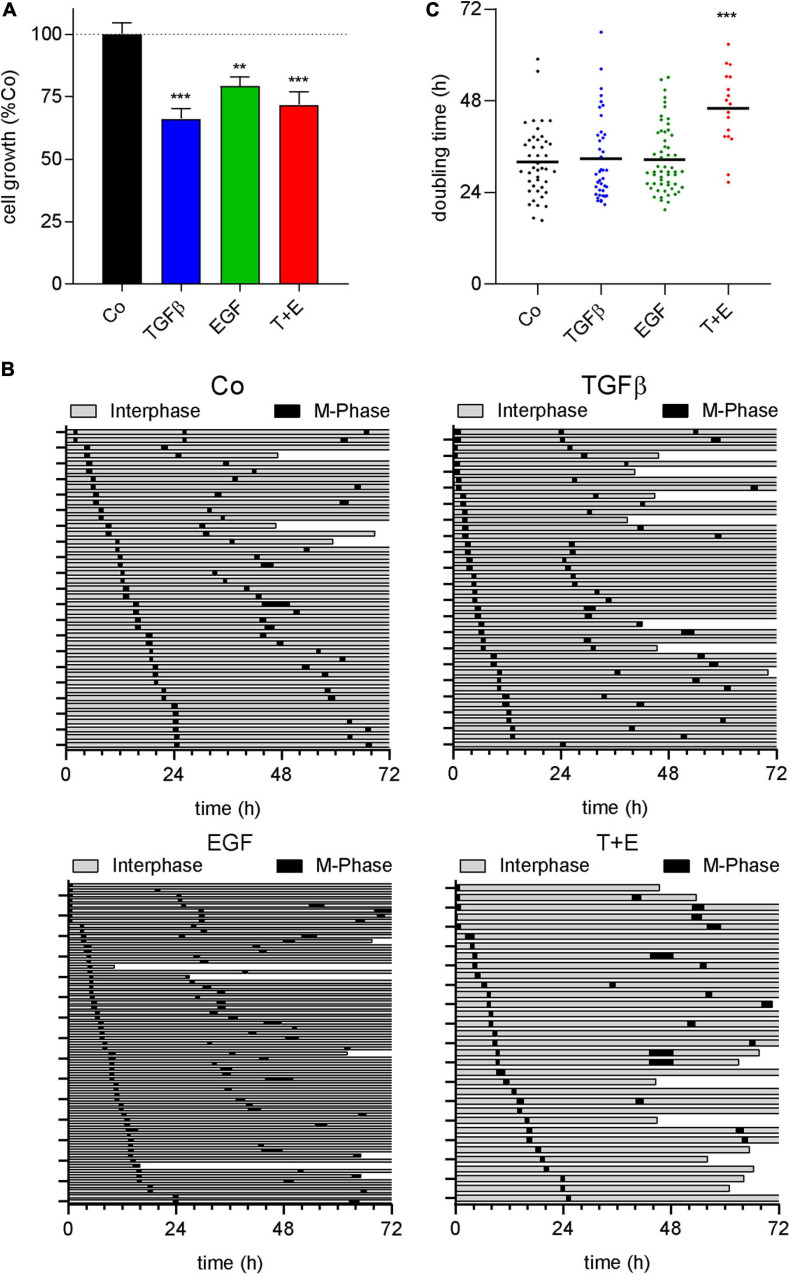
All treatments decrease cell number and the combination increases doubling time. **(A)** Quantification of SYBR green-based cell proliferation assays 72 h after growth factor treatment with TGFβ (5 ng/mL), EGF (50 ng/mL), or a combination of both (T + E) as indicated. Bars represent means and SEM of three experiments. **(B)** Cell fate maps created from videomicroscopy. Each row represents one single cell; the time of interphase is shown in gray, while M-phases are black. The end of a bar before 72 h indicates cell death. **(C)** Doubling time of single cells with treatment as indicated, extracted from cell fate maps. Dots represent individual cell doublings. ***P < 0.001, **P < 0.01, treatment versus control, one-way ANOVA with Dunnett’s multiple comparisons test.

Epidermal growth factor and its receptor are well known for their ability to stimulate cell growth and survival of normal and malignant cells (Sibilia et al., 2007; Cheaito et al., 2020). TGFβ, in contrast, induces cell death in many normal epithelial cell types but can stimulate growth and survival of mesenchymal cells (Zhang Y. et al., 2017). Since the increase in LDHA from the above proteomics data indicated an increased cell death by all treatments, we analyzed growth factor-induced impacts on cell growth, cell cycle progression, and survival. SYBR green-based proliferation assays after treatment with either growth factor or the combination showed a decrease in cell number after 72 h of treatment, which was most pronounced with TGFβ (Figure 6A). Cell fate maps generated from live cell videomicroscopy data (Figure 6B) showed 9.8, 17.5, 11.1, and 36.3% of cell death, respectively, for the control and cultures treated with TGFβ, EGF, and the combination. Regarding cell cycle, no changes in M phase length were observed in any treatment (Supplementary Figure S8). While neither growth factor alone altered the time between cell divisions, a significant increase was observed in the combination-treated group (Figures 6B,C).

### EGF Does Not Contribute to EMT or Invasion Either Alone or in Combination With TGFβ

**FIGURE 7:**
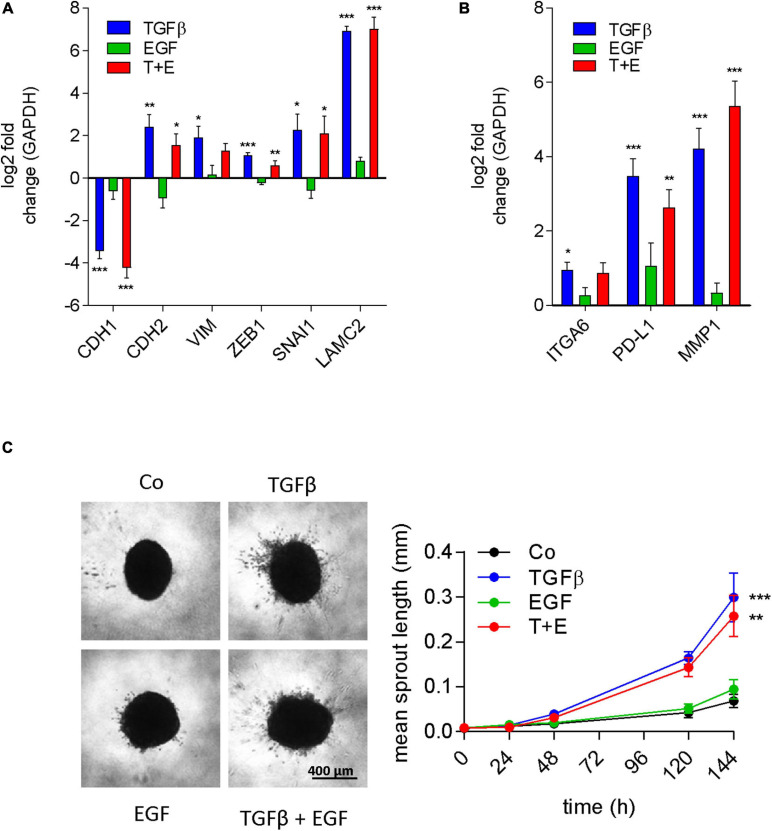
EGF does not contribute to EMT or invasion either alone or in combination with TGFβ. Log2 fold change of gene expression after 24 h of treatment as indicated versus controls of **(A)** the typical EMT marker genes E-cadherin (CDH1), N-cadherin (CDH2), vimentin (VIM), Zeb1, Snail (SNAI1), and laminin γ2 (LAMC2) and **(B)** genes previously linked to EGF-induced EMT [integrin α6 (ITGA6), PD-L1, and MMP1] was determined by qRT-PCR (mean and SEM of three biological repeats performed in duplicates). GAPDH was used as housekeeping gene for normalization. **(C)** Representative images and quantification of invasive sprouts formed by A549 cell spheroids (*n* = 9) embedded in a 3D collagen matrix after treatment with TGFβ, EGF, or both (T + E) as indicated. Data are shown as mean and SEM of nine spheroids derived from three biological repeats. ***P < 0.001, **P < 0.01, *P < 0.05 treatment versus control, one-way ANOVA with Dunnett’s multiple comparisons test.

A549 cells are a well-known model for TGFβ-induced EMT (Kim et al., 2007). The stimulation of migration, the morphological alterations, and the protein expression changes observed in our experiments are all consistent with induction of EMT by TGFβ. EGF has also been described to induce EMT in different model systems like colon cancer (Sakuma et al., 2012) and mesothelioma cell lines (Schelch et al., 2018b), and was reported to cooperate with TGFβ for EMT induction (Docherty et al., 2006; Uttamsingh et al., 2008). Since the proteomics data had revealed a strong connection to EMT for treatments containing TGFβ, but not for EGF treatment, we analyzed mRNA expression changes of classic EMT marker genes in all groups. Indeed, E-cadherin transcript expression was repressed and an increase of N-cadherin, Vimentin, Zeb1, Snail, and Laminin C2 mRNA was seen in cultures treated with TGFβ (Figure 7A). In contrast, EGF had no significant effect on any of these EMT markers on its own. Moreover, there was no significant difference between the combination and TGFβ alone. We then expanded the gene panel to include ITGA6, PD-L1, and MMP1, which were previously shown to be upregulated by EGF in EMT models of mesothelioma (Schelch et al., 2018b). However, none of these genes were significantly affected by EGF treatment in A549 cells (Figure 7B). Interestingly, these genes were significantly upregulated with TGFβ. To test whether these EMT-like expression patterns translated into invasive potential, sprouting assays were conducted. Consistent with the expression of MMP2 and other EMT markers, only TGFβ was able to significantly induce invasive sprouting (Figure 7C). In contrast to its strong effect on cell migration, EGF had no significant effect on spouting either alone or in the combination treatment.

## Discussion

The signaling pathways of EGF and TGFβ both represent relevant targets in several cancers. EGFR is hyperactivated by mutations in about 10% of NSCLC patients (Lynch et al., 2004) and by gene amplification in an even larger fraction of glioblastoma patients (Libermann et al., 1985). Kinase inhibitors of EGFR are used in EGFR mutated lung cancer patients (Lynch et al., 2004), and EGFR targeting monoclonal antibodies are used in colorectal cancer patients in combination with chemotherapy (Eisterer and Prager, 2019). Kinase inhibitors targeting the TGFβ receptor ALK5 have shown promising results in pancreatic cancer and hepatocellular carcinoma in clinical trials (de Gramont et al., 2017). Thus, dissecting the contribution of EGF and TGFβ signals to malignant cell behavior is of substantial interest for therapeutic applications. Under the serum-free conditions used in our experiments, both factors had a strong stimulating effect on migration but repressed cell growth. This would be in line with the “go or grow hypothesis” suggesting that cells will favor either proliferation or migration but not both at the same time (Giese et al., 1996). Although EGF was shown to stimulate cell survival in multiple models (Henson and Gibson, 2006), the lack of serum-contained survival factors in our experiments might result in oncogene-induced cell death, a phenomenon described for activation of both EGFR (Ali et al., 2018) and Ras (Chi et al., 1999).

An interesting finding is the strong stimulation of migration by EGF, despite the presence of oncogenic Ras (*KRAS*^*G*12*S*^) in A549 cells (Pender et al., 2015). Although oncogenic Ras leads to hyperactivation of the MAPK cascade (Takacs et al., 2020), EGF treatment was nevertheless able to further increase Erk phosphorylation suggesting that mutated Ras leads to constitutive but submaximal activation of Erk via the MAPK cascade. Similar to the results presented here, dependency of EGF-stimulated migration on the MAPK pathway was previously reported in mesothelioma cell lines, albeit in the absence of oncogenic Ras (Schelch et al., 2018b). For TGFβ, inhibition of MEK was unable to block the increase in migration and scattering. This is in contrast to previous reports showing crosstalk with Ras and Ras-dependent pathways to be essential to switch the TGβ response from pro-apoptotic to pro-migratory (Grusch et al., 2010).

Our data suggest that EGF-induced and TGFβ-induced pro-migratory effects in A549 cells are more independent of each other than previously reported. For instance, in the lung cancer cell lines H322 and the pancreatic cancer cell lines HPAF-II, EGF and TGFβ cooperated in the induction of EMT (Buonato et al., 2015). Although multiple reports show induction of EMT by EGF or other growth factors like FGF2 or HGF binding to and activating receptor tyrosine kinases (Sakuma et al., 2012; Farrell et al., 2014; Schelch et al., 2018b), EGF-induced migration in A549 cells was not connected to an increase in EMT. This was suggested by both proteomics and mRNA data and further confirmed by the inability to stimulate invasive behavior. EGF was previously shown to regulate the EMT markers E-cadherin and Vimentin in mesothelioma cells (Schelch et al., 2018b) and colorectal cancer cells (Sakuma et al., 2012) but failed to do so in A549 cells. TGFβ, in contrast, showed a strong repression of E-cadherin and stimulation of Vimentin in A549 cells. Moreover, several factors, including the calcium-binding protein Calreticulin (Wu et al., 2017), the transcriptional coregulator Ski (Yang et al., 2015), and the microRNA miR-205 (Zeng et al., 2016), have previously been shown to either promote or repress TGFβ-induced EMT in A549 cells by interfering with various components of the TGFβ signaling axis.

Despite the differences with respect to EMT induction, cell shape changes, and migration kinetics, there was a considerable overlap in protein expression changes between EGF and TGFβ. This is best exemplified by the far higher numbers of proteins changed in the same than in the opposite direction by both treatments. It is likely that at least some of these proteins are required for cell motility irrespective of the stimulus. Additionally, the ability of the ROCK inhibitor to block the scattering induced by both EGF and TGFβ suggests the dependency of both growth factors on an overlapping set of motility proteins. ROCK1 and ROCK2 are central regulators of actin-myosin contractility and actin cytoskeleton dynamics and are being discussed as potential therapy targets in cancer and other diseases (Shahbazi et al., 2020). The lack of an effect of EGF on matrix degrading enzymes like MMP2 and the specific morphology of EGF stimulated cells could indicate that EGF may favor a more amoeboid over a mesenchymal type of cell migration compared to TGFβ. In the proteomics data, however, the GO term amoeboid migration was not differently represented between EGF-treated and TGFβ-treated A549 cells. Moreover, in the breast cancer cell line MDA-MB-231, EGF was shown to promote mesenchymal rather than amoeboid migration (Geum et al., 2016).

With respect to clinical implications, our data suggest that stimulation of EGFR by EGF or other ligands of this receptor may have additional pro-tumorigenic effects such as increased cell migration, even in the presence of Ras mutations. Coming from a single cell model, these data have to be interpreted with caution. In the clinic, treatment of Ras mutated tumors with EGFR-targeting agents is generally not thought to be effective (Ghimessy et al., 2020). In line with our findings, however, recent data from *in vivo* models likewise suggest that EGFR contributes to lung tumorigenesis in the presence of mutated Ras (Moll et al., 2018). On the other hand, blocking EGFR may only have limited effects on invasion and may not mitigate TGFβ-induced EMT. Indeed, occurrence of EMT has been observed in NSCLC patients as a means of acquired resistance under therapy with EGFR inhibitors (Zhu et al., 2019). Based on the data from A549 cells, blocking TGFβ signals could be more effective in combating invasion and EMT at least in a subset of lung cancer patients, but would still allow for EGF induced migration. In terms of inhibiting cell migration and in consequence spread of tumor cells, a combination approach of EGFR and TGFβ-receptor inhibitors could be more effective. Preclinical studies using agent combinations targeting both EGFR and TGFβ-receptors have shown enhanced antitumor effects (Bedi et al., 2012; Serizawa et al., 2013), and our data support further research in this direction.

## Data Availability Statement

The mass spectrometry proteomics data have been deposited to the ProteomeXchange Consortium via the PRIDE (Perez-Riverol et al., 2019) partner repository with the dataset identifier PXD023024 and 10.6019/PXD02302.

## Author Contributions

KS, LV, AS, JB, and RM performed the experiments. TM, AS, CG, and MG coordinated the study and supervised all experiments. KS, AS, and MG wrote the manuscript. All authors discussed the results, commented on the manuscript, and approved the submitted version.

## Conflict of Interest

The authors declare that the research was conducted in the absence of any commercial or financial relationships that could be construed as a potential conflict of interest.

## Footnotes

1 https://bioinfogp.cnb.csic.es/tools/venny/index.html

2 https://software.broadinstitute.org/morpheus

3 https://www.gsea-msigdb.org/gsea/msigdb

## Supplementary Material

Supplementary Figure 1 Representative confocal images (individual channels and merged) of A549 cells 48 h after treatment with EGF, TGFβ, or a combination of both (T + E) as indicated. Cells were fixed and immunocytochemically stained with phalloidin (actin, red), DAPI (nucleus, blue), and an antibody against tubulin (green).

Supplementary Figure 2 Immunoblots of A549 cells treated with EGF for 30 min in the presence or absence of the MEK inhibitor U0126. A representative example and pErk/Erk ratios from three biological repeats are shown. Beta actin was used as a control for equal sample loading.

Supplementary Figure 3 Venn diagrams showing the number of upregulated and downregulated proteins (q < 0.25) in the nuclear fraction **(A)** and the supernatant **(B)** of A549 cells 48 h after treatment with TGFβ, EGF, or a combination of both (T + E) compared to vehicle-treated controls.

Supplementary Figure 4 Heatmap and unsupervised clustering of the mean LFQ values of all detected proteins in the cytoplasma (CYT), the nucleus (NE), and the supernatant (SUP) of A549 cells 48 h after the respective treatments.

Supplementary Figure 5 Heatmap showing expression levels of selected migration-related proteins **(A)** in the cytoplasmic fraction and **(B)** in the supernatant [LFQ values, * indicates q < 0.25 compared to control (Co)].

Supplementary Figure 6 Proteins in the cytoplasmic fraction of A549 cells upregulated (q < 0.25) by TGFβ but not EGF (upper panel), EGF but not TGFβ (middle panel), or the combination treatment but not either growth factor alone (lower panel), were subjected to GO term analysis. The top five biological process categories ranked by *p*-value are shown.

Supplementary Figure 7 The up- and downregulated cell motility proteins from all three cell fractions of A549 cells treated with TGFβ (upper panel) and EGF (lower panel) were pooled and subjected to GO term analysis. The top five biological process categories ranked by *p*-value are shown.

Supplementary Figure 8 M-phase length of single cells with treatment as indicated, extracted from cell fate maps.

Supplementary Table 1 Primers used for PCR.

Supplementary Table 2 Identified proteins by proteome analyses.

Supplementary Table 3 Proteins upregulated and downregulated (q < 0.25) in the cytoplasmic, supernatant, and nuclear fractions of A549 cells treated with EGF, TGFβ, or the combination versus untreated controls.

Supplementary Table 4 Number of proteins (i) up- and downregulated (q < 0.25) in the cytoplasmic (cyt) fractions of A549 cells treated with TGFβ, EGF, or both (E + T) and (ii) number of overlapping proteins in these fractions.

Supplementary Table 5 Number of proteins (i) up- and downregulated (q < 0.25) in the nuclear (nuc) fractions of A549 cells treated with TGFβ, EGF, or both (E + T) and (ii) number of overlapping proteins in these fractions.

Supplementary Table 6 Number of proteins (i) up- and downregulated (q < 0.25) in the supernatant (sup) fractions of A549 cells treated with TGFβ, EGF, or both (E + T) and (ii) number of overlapping proteins in these fractions.

Supplementary Video 1 Videomicroscopy of A549 cells treated with EGF, TGFβ, EGF + TGFβ, and vehicle only (Co) over the course of 72 h.

Supplementary Data Scans of uncropped immunoblots.
