# Supplementary material for: EGF Induces Migration Independent of EMT or Invasion in A549 Lung Adenocarcinoma Cells
Source: Front Cell Dev Biol. 2021 Mar 12;9:634371. doi: 10.3389/fcell.2021.634371 (PMC7994520; doi:10.3389/fcell.2021.634371)
Supplement: Supplementary Figure 1 — Representative confocal images (individual channels and merged) of A549 cells 48 h after treatment with EGF, TGFβ, or a combination of both (T + E) as indicated. Cells were fixed and immunocytochemically stained with phalloidin (actin, red), DAPI (nucleus, blue), and an antibody against tubulin (green). [file Data_Sheet_1.PDF]

Control

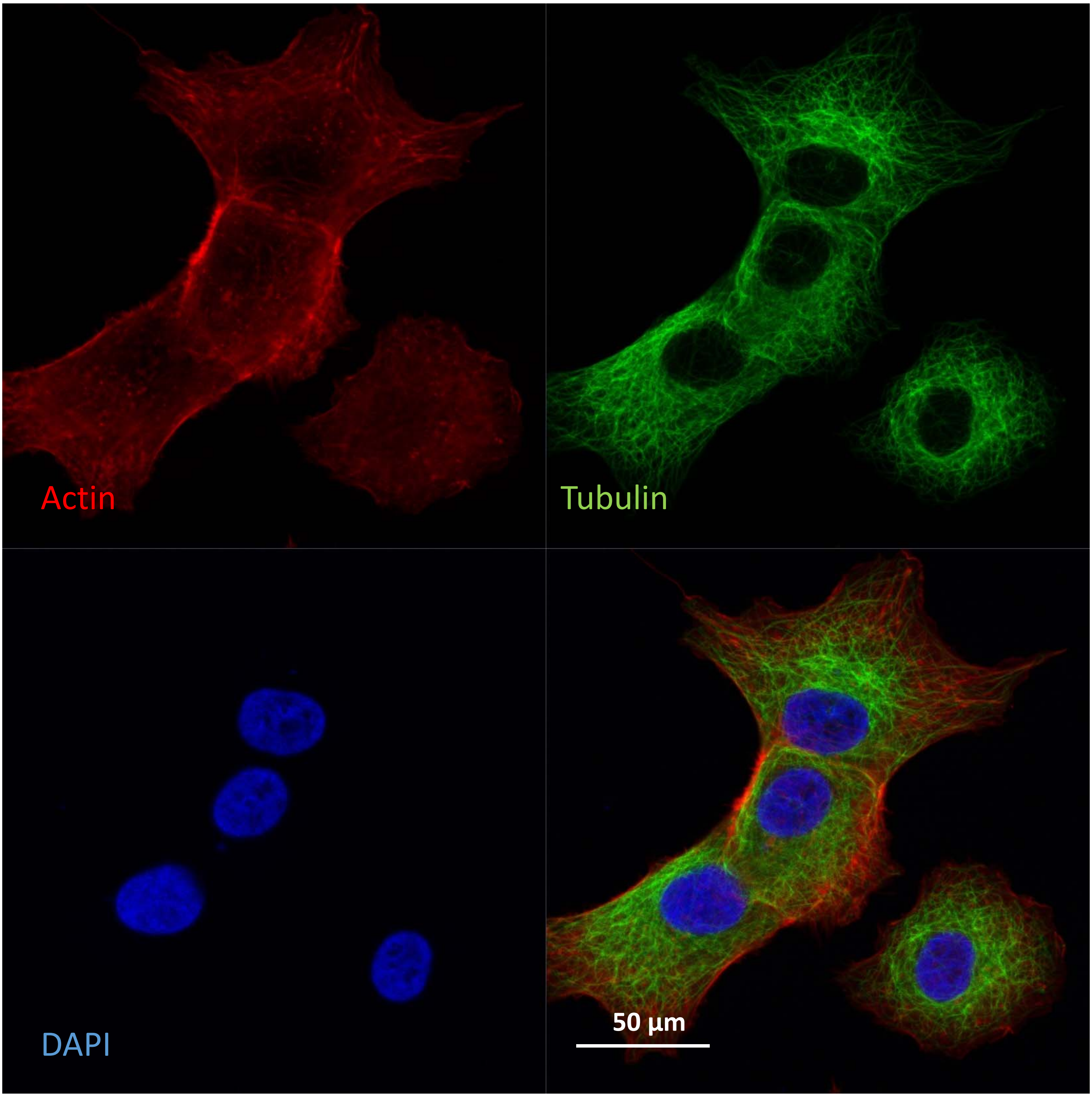


Schelch et al. Figure S1A

TGFβ

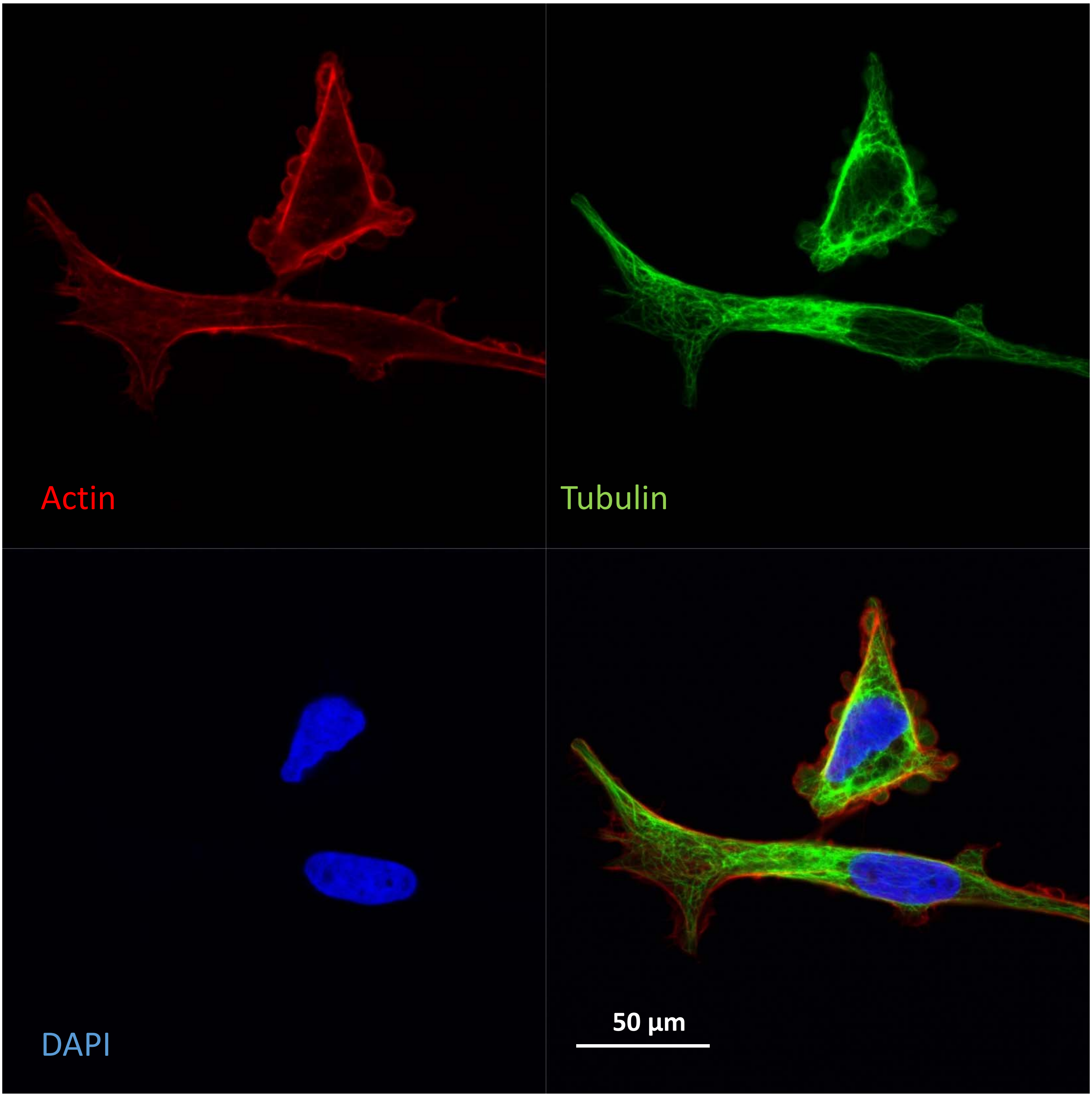


Schelch et al. Figure S1B

EGF

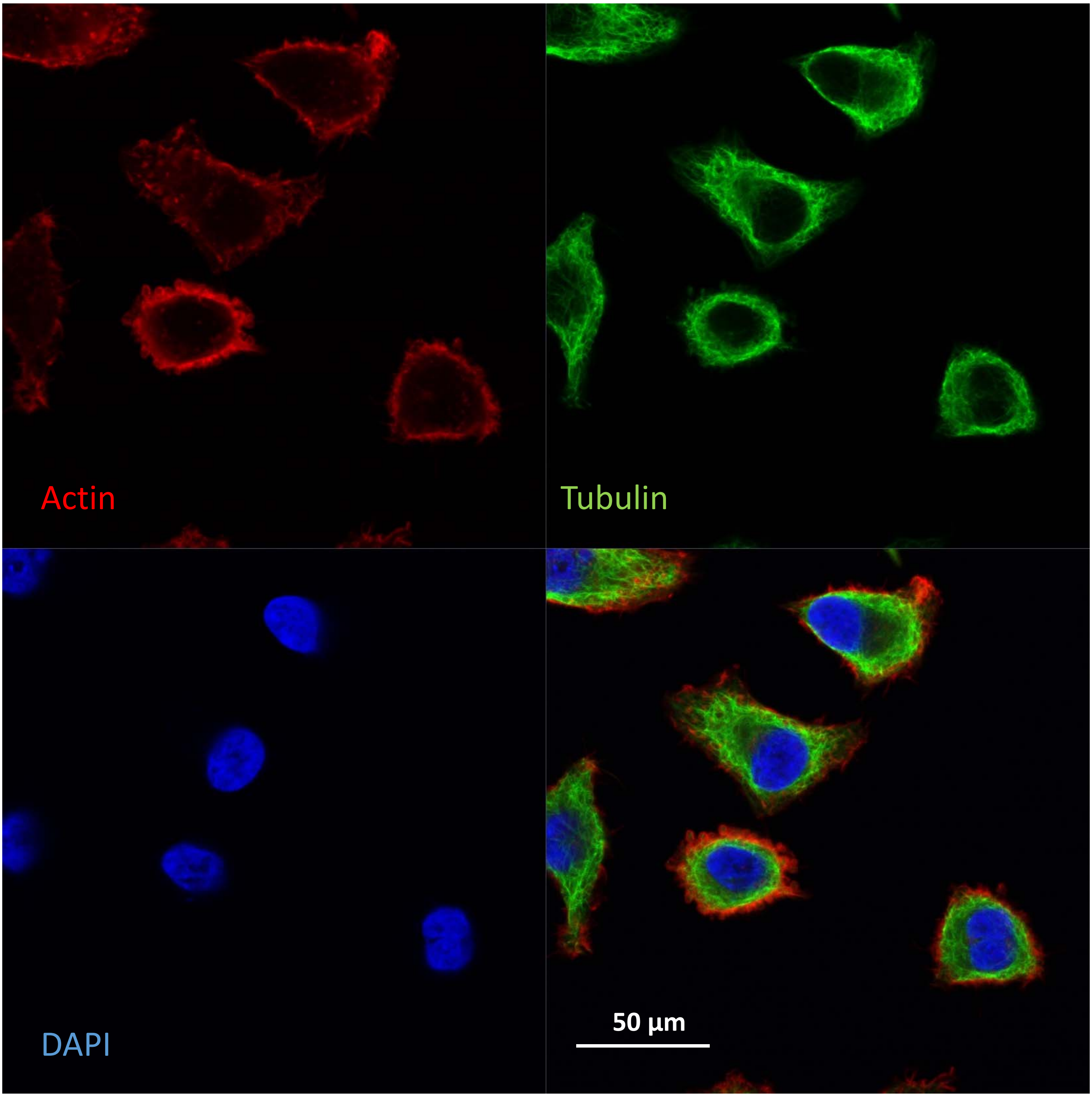


Schelch et al. Figure S1C

T+E

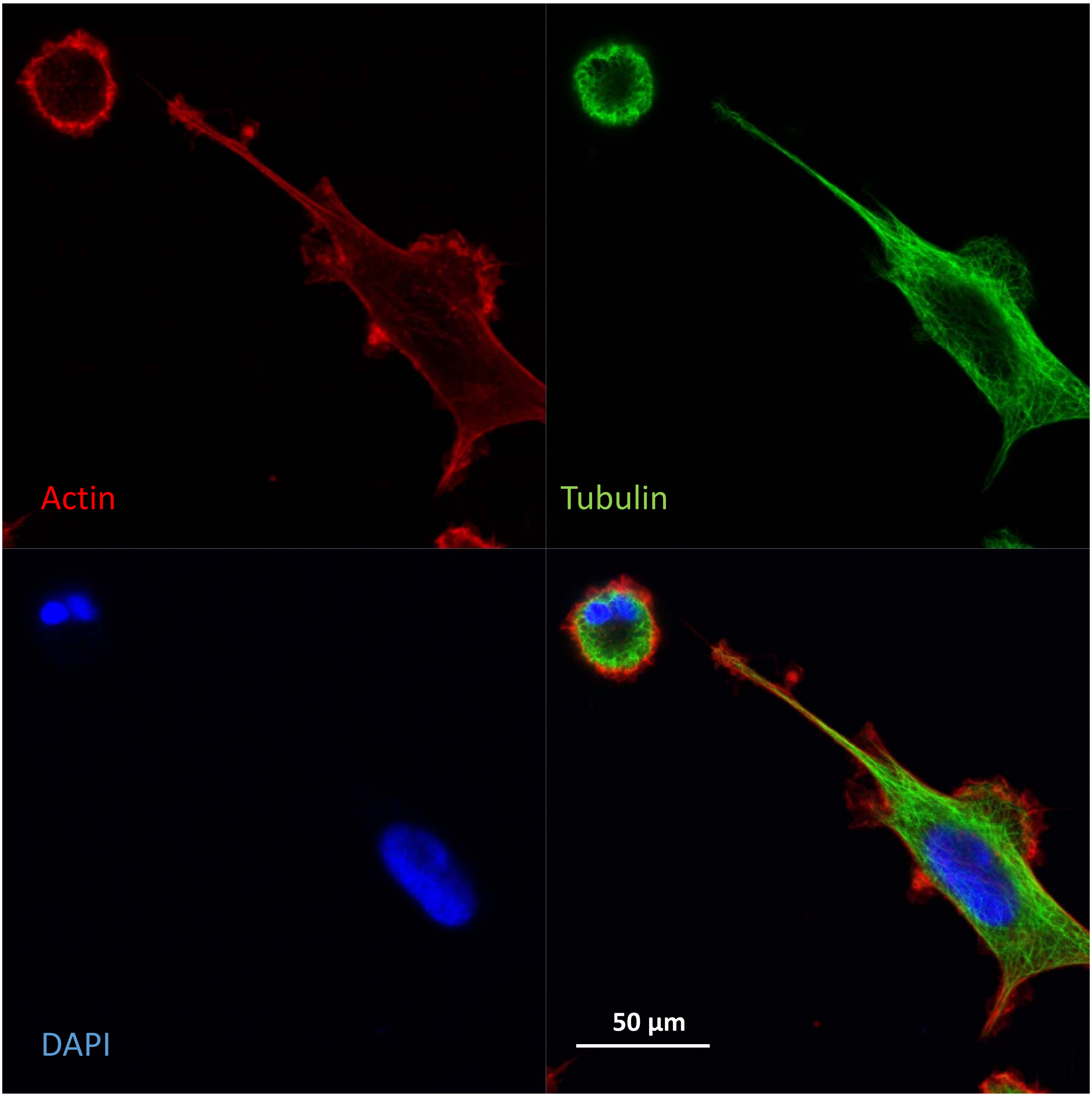


**Figure S1 A-D:** Representative confocal images (individual channels and merged) of A549 cells 48 h after treatment with EGF, TGFβ or a combination of both (T+E) as indicated. Cells were fixed and immunocytochemically stained with phalloidin (actin, red), DAPI (nucleus, blue) and an antibody against tubulin (green).

Schelch et al. Figure S1D

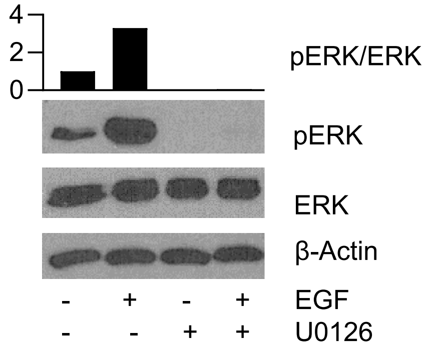


**Figure S2 |** Immunoblots of A549 cells treated with EGF for 30 min in the presence or absence of the MEK inhibitor U0126. A representative example and mean pERK/ERK ratios normalized to control from triplicate experiments are shown. Beta actin was used as a control for equal sample loading. Uncropped immunoblots of all 3 replicates are shown as Supplementary Data.

Schelch et al. Figure S2

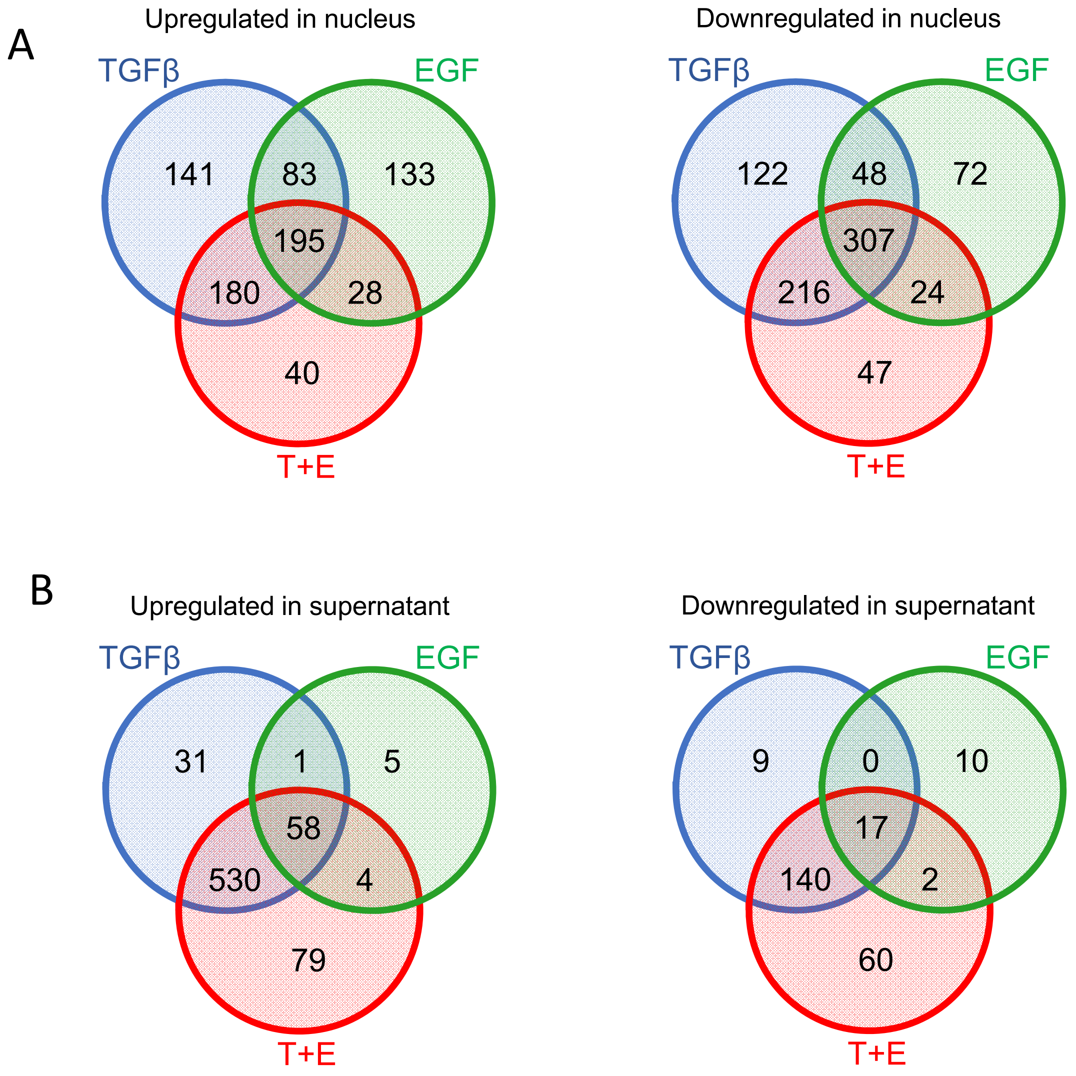


**Figure S3 |** Venn diagrams showing the number of upregulated and downregulated proteins (q <0.25) in the nuclear fraction (A) and the supernatant (B) of A549 cells 48 h after treatment with TGFβ, EGF or a combination of both (T+E) compared to vehicle-treated controls.

Schelch et al. Figure S3

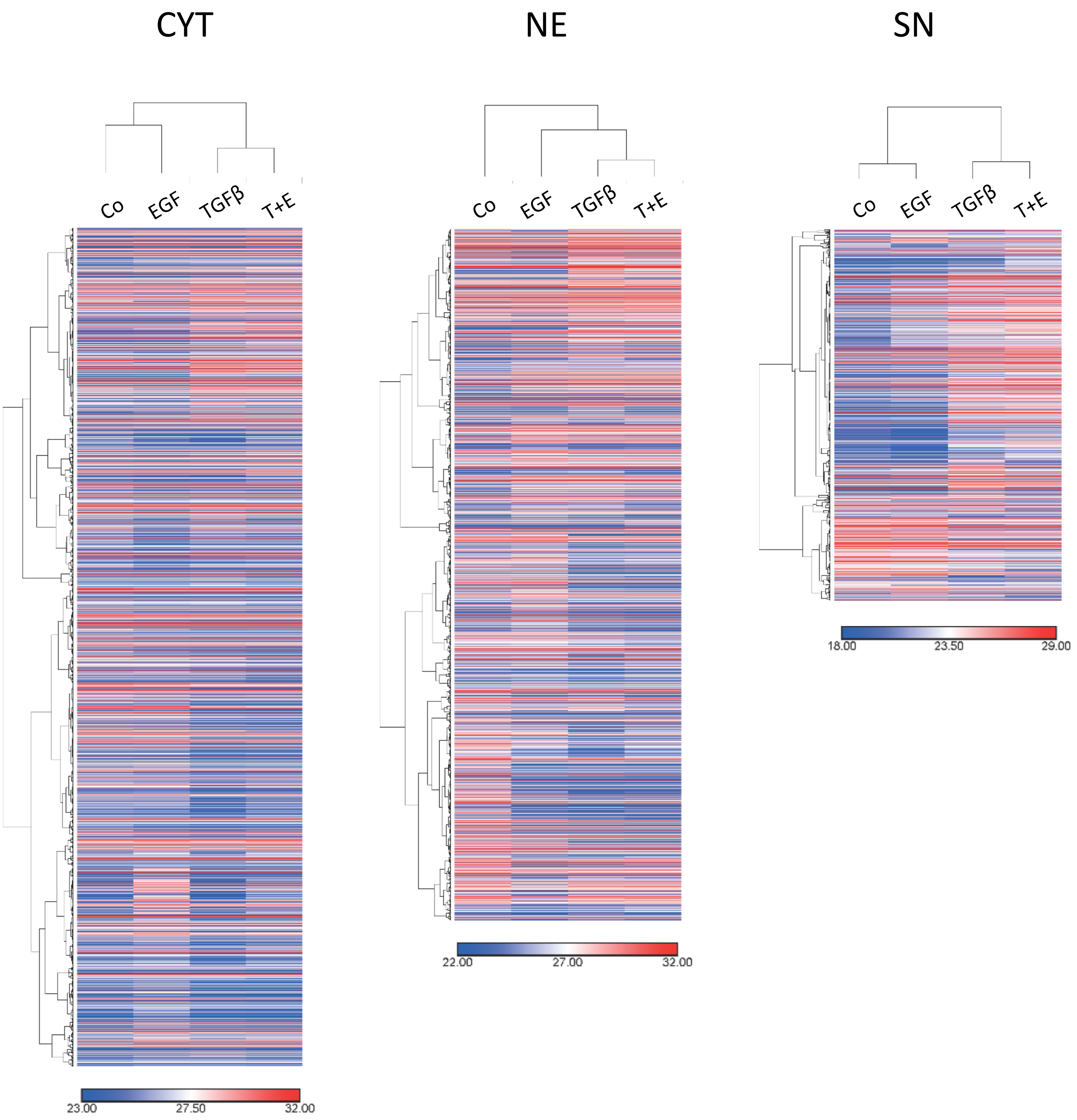


**Figure S4 |** Heatmap and unsupervised clustering of the mean LFQ values of all detected proteins in the cytoplasma (CYT), the nucelus (NE) and the supernatant (SUP) of A549 cells 48 h after the respective treatments.

Schelch et al. Figure S4

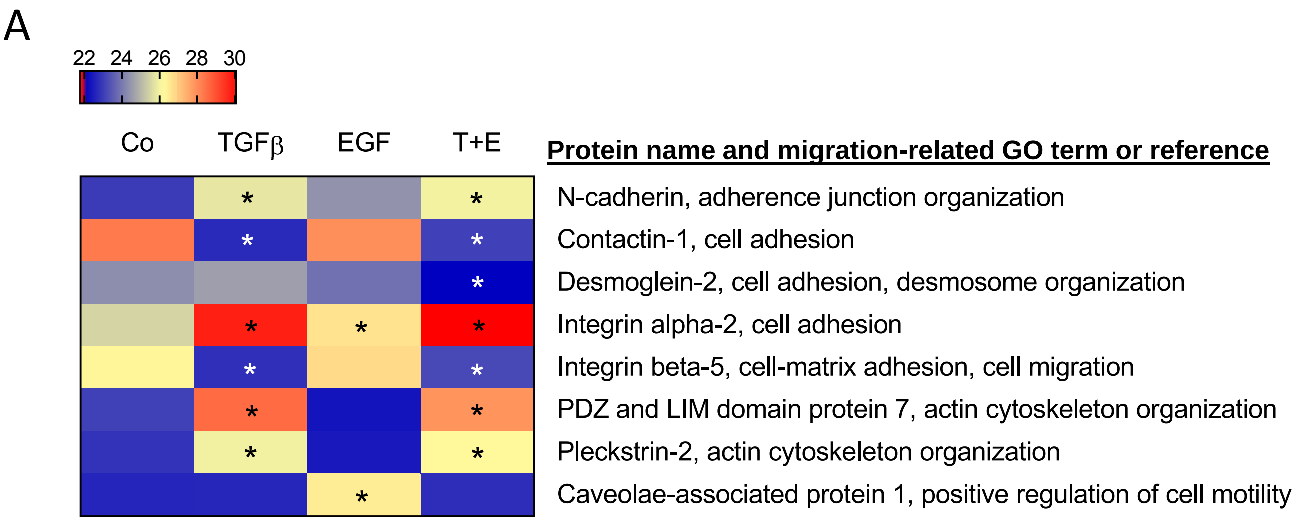


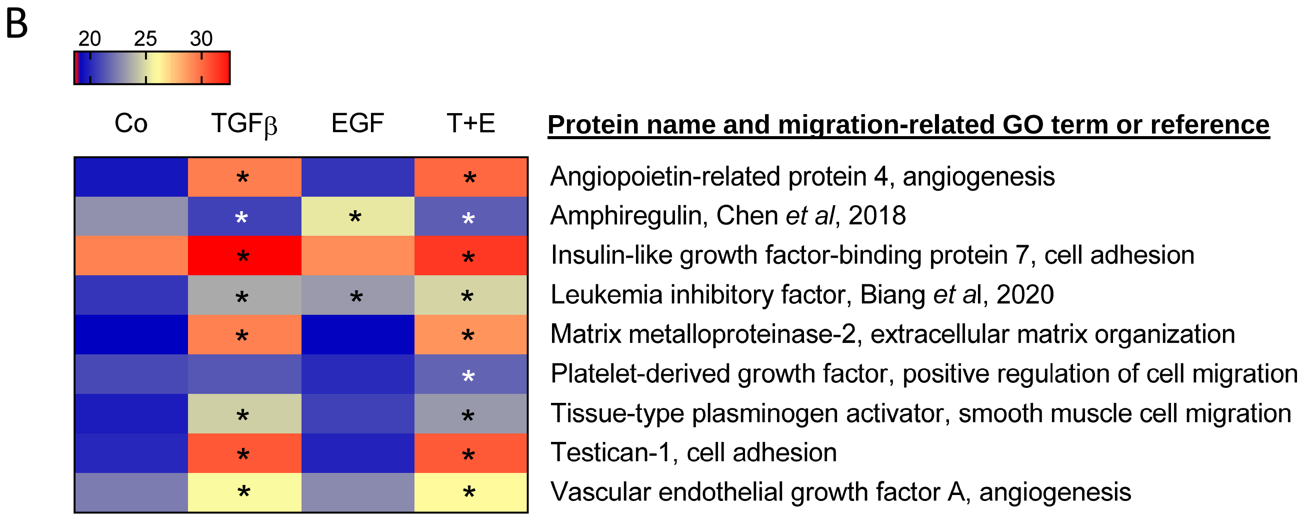


**Figure S5 |** Heatmap showing expression levels of selected migration-related proteins (A) in the cytoplasmic fraction and (B) in the supernatant (LFQ values, * indicates q <0.25 compared to control (Co)).

**References in Figure S5:**

Chen, J. C., Huang C., Lee I. N., Wu Y. P. and Tang C. H. (2018). Amphiregulin enhances cell migration and resistance to doxorubicin in chondrosarcoma cells through the MAPK pathway. *Mol.Carcinog.* 57, 1816-1824. doi: 10.1002/mc.22899

Bian, S. B., Yang Y., Liang W. Q., Zhang K. C., Chen L. and Zhang Z. T. (2020). Leukemia inhibitory factor promotes gastric cancer cell proliferation, migration, and invasion via the LIFR-Hippo-YAP pathway. *Ann. N. Y. Acad. Sci.* doi: 10.1111/nyas.14466

Schelch et al. Figure S5

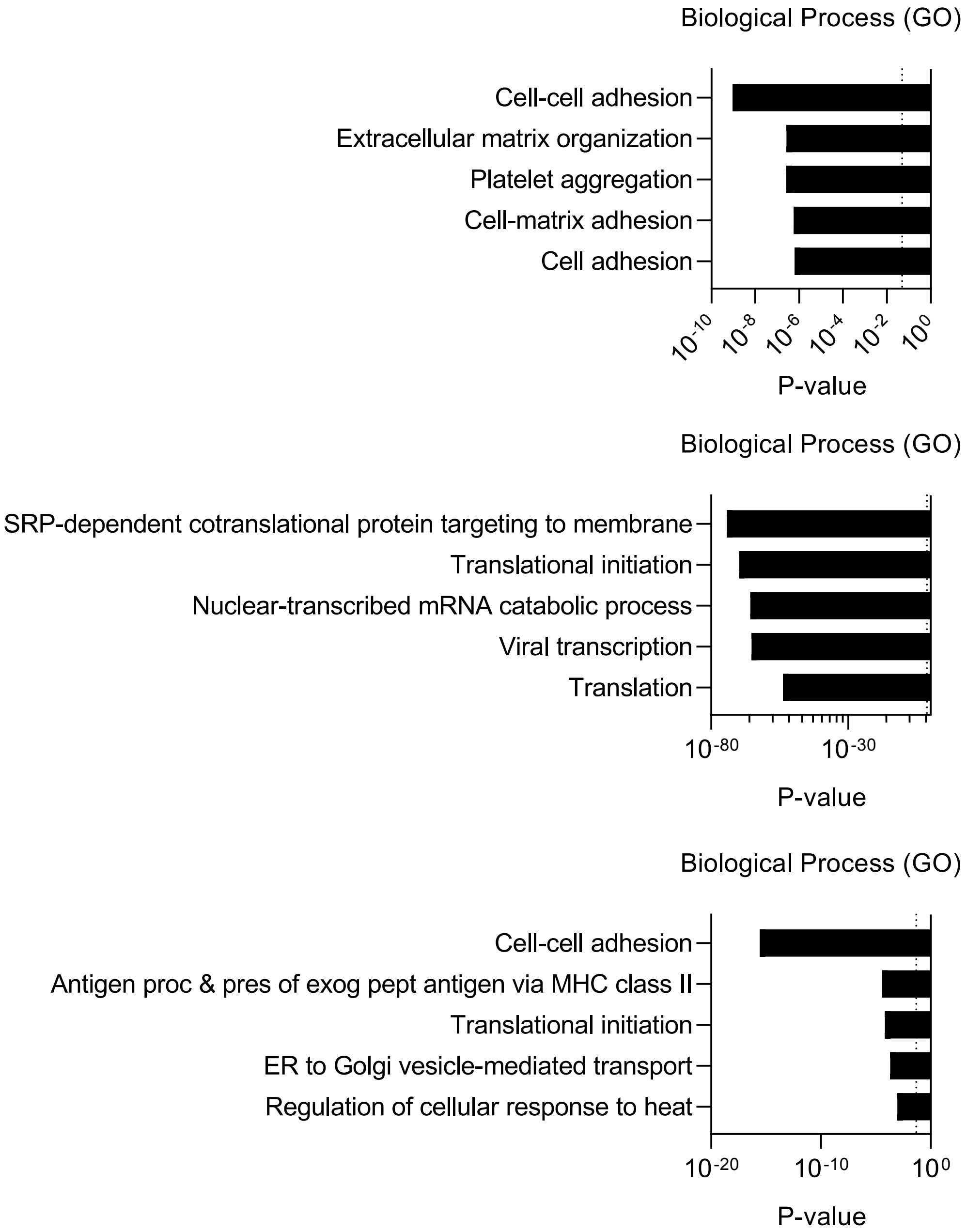


**Figure S6 |** Proteins in the cytoplasmic fraction of A549 cells upregulated ($q < 0.25$) by TGFβ but not EGF (upper panel), EGF but not TGFβ (middle panel) or the combination treatment but not either growth factor alone (lower panel) were subjected to GO term analysis and the top five biological process categories ranked by p value are shown.

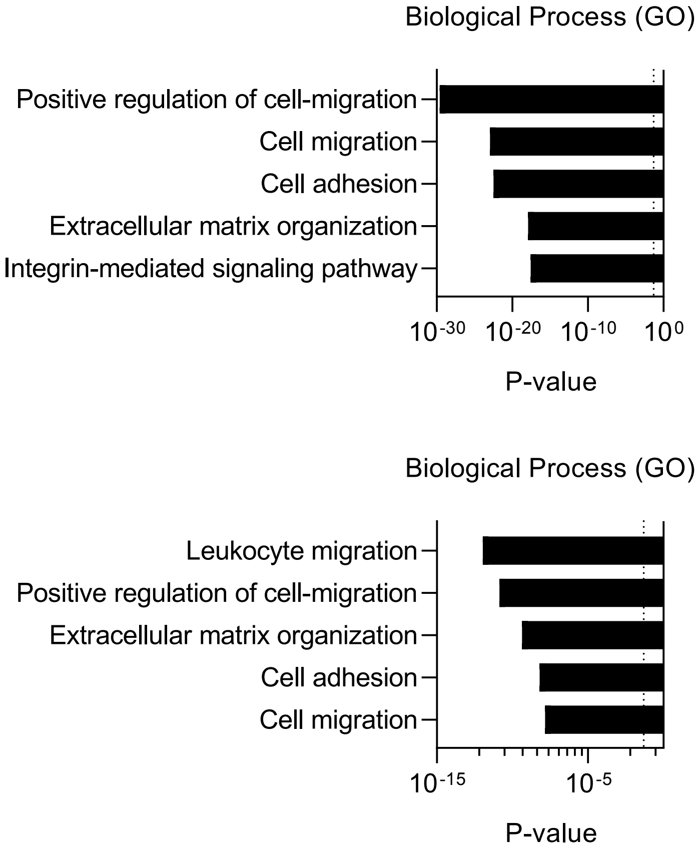


**Figure S7 |** The up- and downregulated cell motility proteins from all three cell fractions of A549 cells treated with TGFβ (upper panel) and EGF (lower panel) were pooled and subjected to GO term analysis. The top five biological process categories ranked by p value are shown.

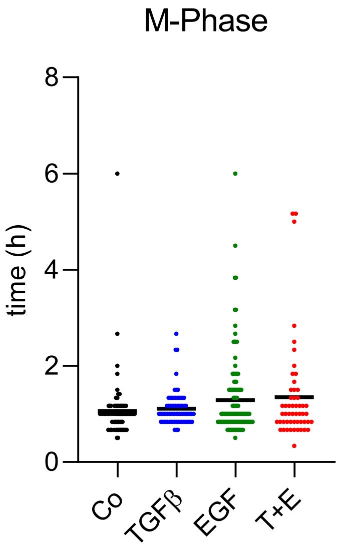


**Figure S8 |** M-phase length of single cells with treatment as indicated, extracted from cell fate maps. Dots represent individual M-phases. No statistically significant changes in treated samples versus control were observed (One-way ANOVA with Dunnett´s multiple comparisons test).
