# Supplementary material for: EGF Induces Migration Independent of EMT or Invasion in A549 Lung Adenocarcinoma Cells
Source: Front Cell Dev Biol. 2021 Mar 12;9:634371. doi: 10.3389/fcell.2021.634371 (PMC7994520; doi:10.3389/fcell.2021.634371)
Supplement: Supplementary Table 1 — Primers used for PCR. [file Data_Sheet_2.PDF]

Supplementary Table S1| Primers used for PCR

oligonucleotide name	sequence (5´-3´)
CDH1 for	CAGAGCCTCTGGATAGAGAACGCA
CDH1 rev	GGCATTGTAGGTGTTCACATCATCGTC
CDH2 for	GCATCATCATCCTGCTTATCC
CDH2 rev	TTCTCCTCCACCTTCTTCATC
GAPDH for	AGCTCACTGGCATGGCCTTC
GAPDH rev	ACGCCTGCTTCACCACCTTC
ITGA6 for	GCTCCCAGAGCCAATCAC
ITGA6 rev	CACCGCCACATCATAGCC
LAMC2 for	AGCCAAGAGAACAGCTACC
LAMC2 rev	GTGATGAGCCTGTGAGTATCC
MMP1 for	TACATGCGCACAAATCCC
MMP1 rev	ACAGCCCAGTACTTATTCCC
PD-L1 for	CACCACCAATTCCAAGAG
PD-L1 rev	CTGGGATGACCAATTCAG
SNAI1 for	TATGCTGCCTTCCCAGGCTTG
SNAI1 rev	ATGTGCATCTTGAGGGCACCC
VIM for	GGCTCAGATTCAGGAACAGC
VIM rev	CTGAATCTCATCCTGCAGGC
ZEB1 for	CCAGTGGTCATGATGAAAATGGAACACC
ZEB1 rev	CAGACTGCGTCACATGTCTTTGATCTC
