# Supplementary material for: EGF Induces Migration Independent of EMT or Invasion in A549 Lung Adenocarcinoma Cells
Source: Front Cell Dev Biol. 2021 Mar 12;9:634371. doi: 10.3389/fcell.2021.634371 (PMC7994520; doi:10.3389/fcell.2021.634371)
Supplement: Supplementary Table 4 — Number of proteins (i) up- and downregulated (q < 0.25) in the cytoplasmic (cyt) fractions of A549 cells treated with TGFβ, EGF, or both (E + T) and (ii) number of overlapping proteins in these fractions. [file Data_Sheet_3.PDF]

Supplementary Table S4 | Number of proteins (i) up- and downregulated (q < 0.25) in the cytoplasmic (cyt) fractions of A549 cells treated with TGFβ, EGF or both (E +T) and (ii) number of overlapping proteins in these fractions.

**cyt**	$TGFβ^{up}$	$TGFβ^{down}$	EGF^{up}	EGF^{down}	$E+T^{up}$	$E+T^{down}$
$TGFβ^{up}$	230					
$TGFβ^{down}$	0	378				
EGF^{up}	34	23	212			
EGF^{down}	16	59	0	152		
$E+T^{up}$	189	5	94	17	435	
$E+T^{down}$	1	289	12	85	0	532

Supplementary Table S5 | Number of proteins (i) up- and downregulated (q < 0.25) in the nuclear (nuc) fractions of A549 cells treated with TGFβ, EGF or both (E +T) and (ii) number of overlapping proteins in these fractions.

**nuc**	$TGFβ^{up}$	$TGFβ^{down}$	EGF^{up}	EGF^{down}	$E+T^{up}$	$E+T^{down}$
$TGFβ^{up}$	599					
$TGFβ^{down}$	0	693				
EGF^{up}	278	33	439			
EGF^{down}	22	355	0	451		
$E+T^{up}$	375	5	223	19	443	
$E+T^{down}$	3	523	30	331	0	594

Supplementary Table S6 | Number of proteins (i) up- and downregulated ($q < 0.25$) in the supernatant (sup) fractions of A549 cells treated with TGFβ, EGF or both (E +T) and (ii) number of overlapping proteins in these fractions.

**sup**	$TGFβ^{up}$	$TGFβ^{down}$	EGF^{up}	EGF^{down}	$E+T^{up}$	$E+T^{down}$
$TGFβ^{up}$	620					
$TGFβ^{down}$	0	166				
EGF^{up}	59	4	68			
EGF^{down}	8	17	0	29		
$E+T^{up}$	588	0	62	8	671	
$E+T^{down}$	4	157	3	19	0	219
