# Supplementary material for: EGF Induces Migration Independent of EMT or Invasion in A549 Lung Adenocarcinoma Cells
Source: Front Cell Dev Biol. 2021 Mar 12;9:634371. doi: 10.3389/fcell.2021.634371 (PMC7994520; doi:10.3389/fcell.2021.634371)
Supplement: Supplementary Data — Scans of uncropped immunoblots. [file Data_Sheet_4.PDF]

Figure 3A:

pAKT
30 min

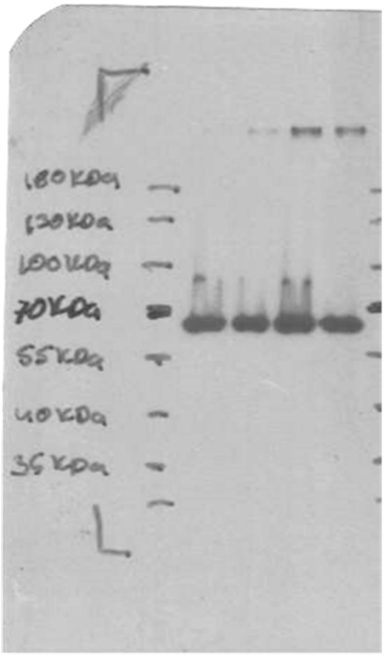

-	+	-	+	TGFβ
-	-	+	+	EGF

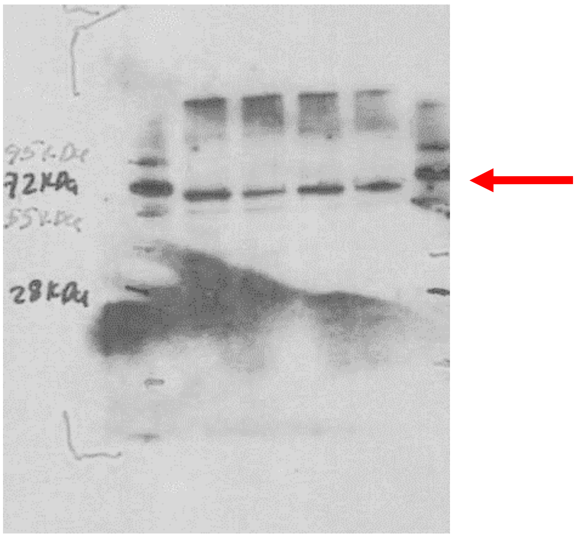

-	+	-	+	TGFβ
-	-	+	+	EGF

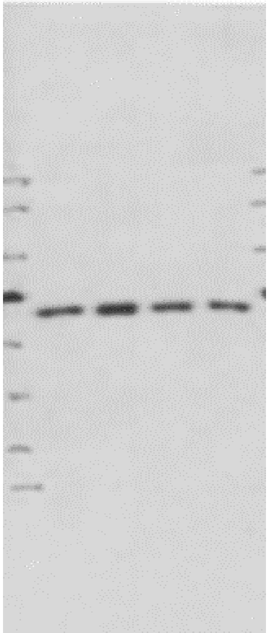

-	+	-	+	TGFβ
-	-	+	+	EGF

Figure 3A:

AKT
30 min

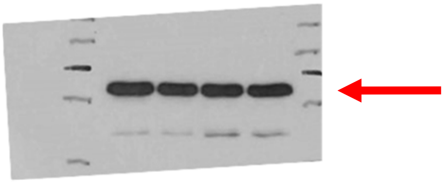

\- + - + TGFβ

\- - + + EGF

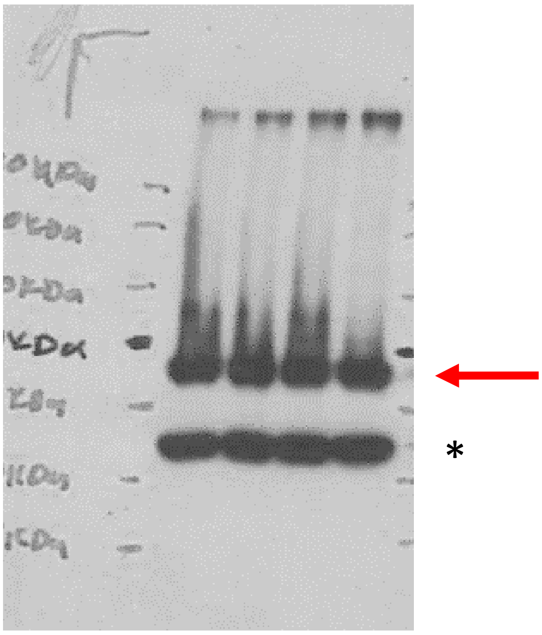

*

\- + - + TGFβ

\- - + + EGF

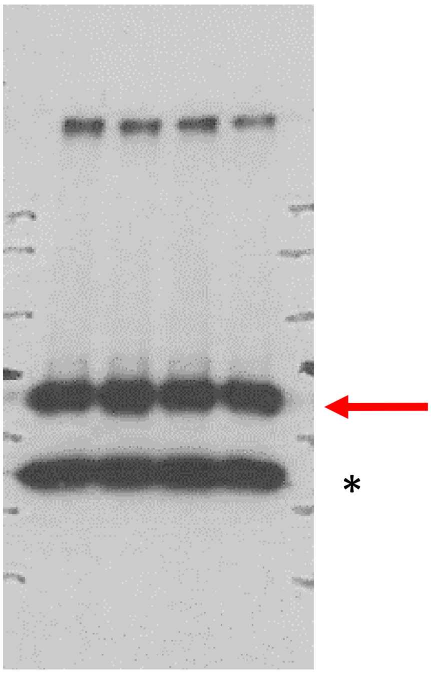

*

\- + - + TGFβ

\- - + + EGF

* Bands from other antibodies used on the same membrane or unspecific bands

Figure 3A:

pERK
30 min

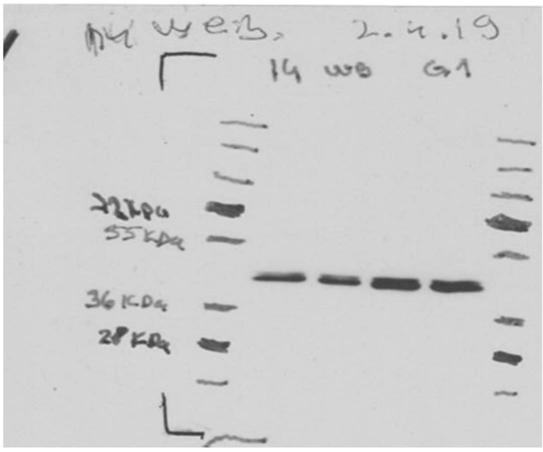

- + - + TGFβ
- - + + EGF

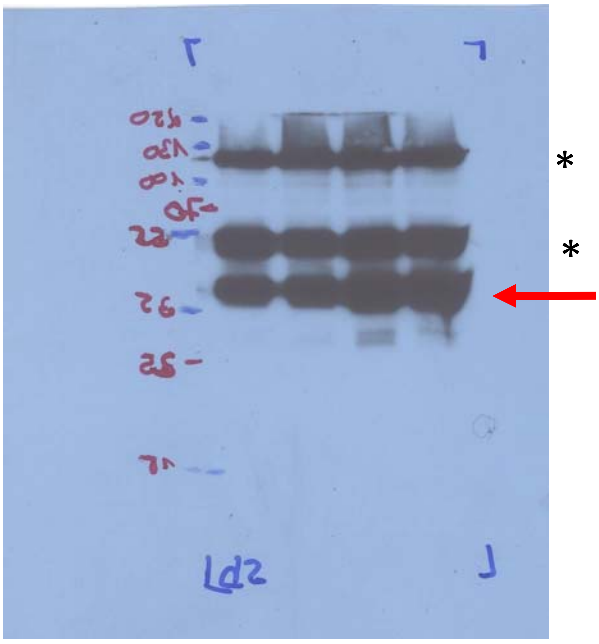

- + - + TGFβ
- - + + EGF

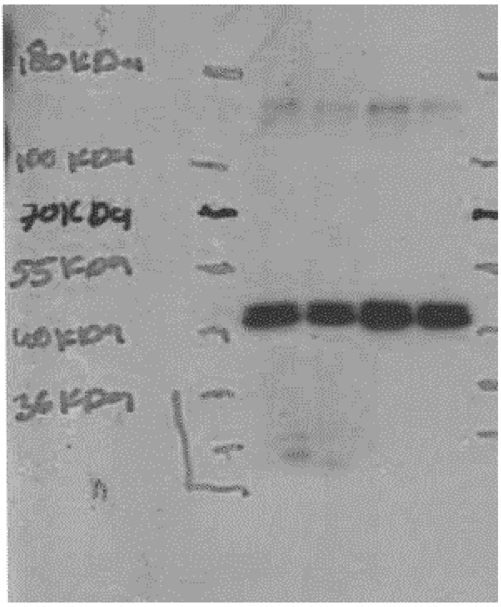

- + - + TGFβ
- - + + EGF

* Bands from other antibodies used on the same membrane or unspecific bands

Figure 3A:

ERK
30 min

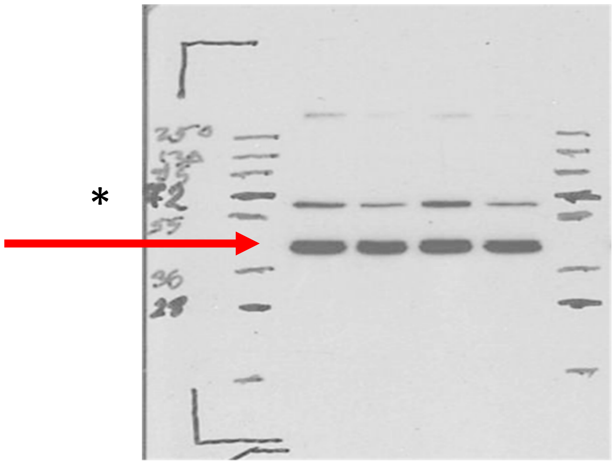

- + - + TGFβ
- - + + EGF

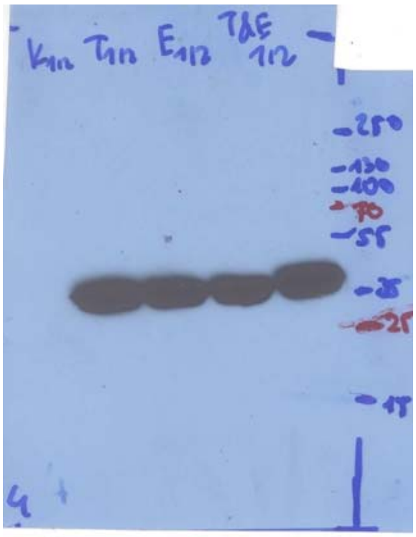

- + - + TGFβ
- - + + EGF

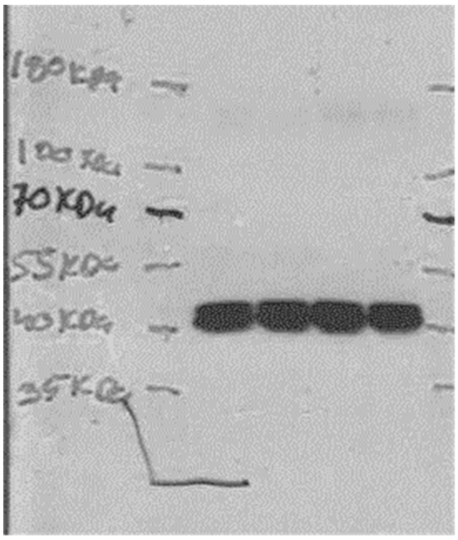

- + - + TGFβ
- - + + EGF

* Bands from other antibodies used on the same membrane or unspecific bands

Figure 3A:

pSMAD2
30 min

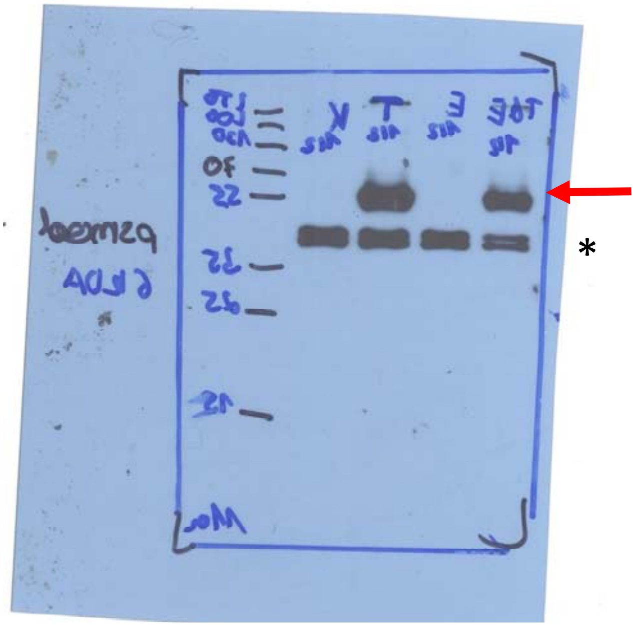

-   +   -   +   TGFβ
-   -   +   +   EGF

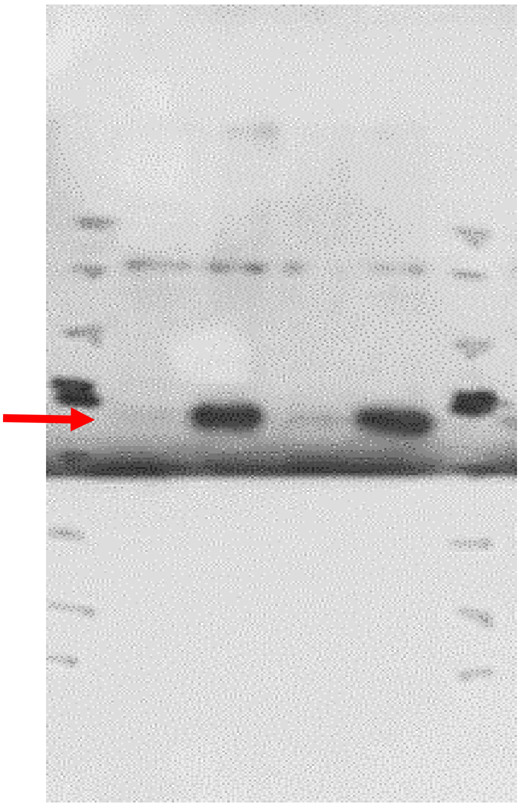

-   +   -   +   TGFβ
-   -   +   +   EGF

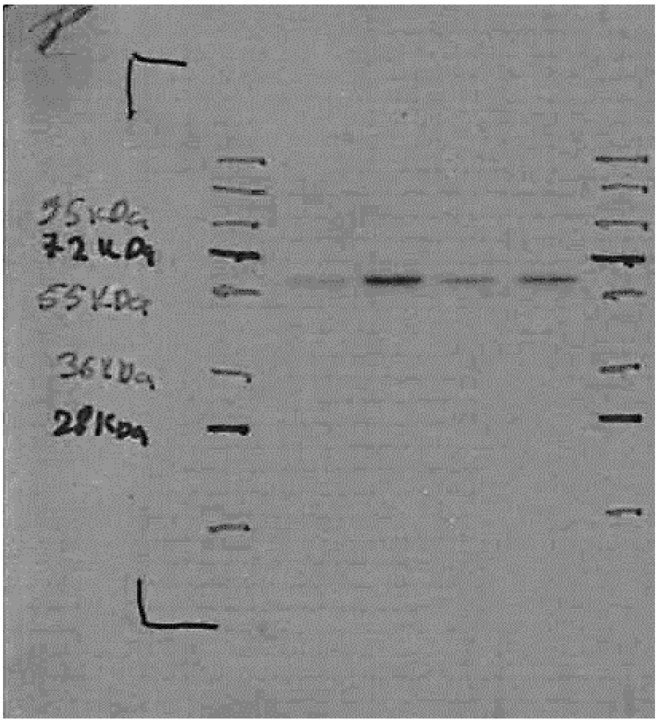

-   +   -   +   TGFβ
-   -   +   +   EGF

* Bands from other antibodies used on the same membrane or unspecific bands

Figure 3A:

SMAD2
30 min

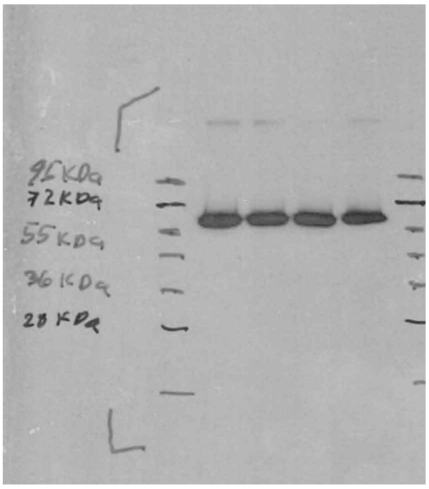

- + - + TGFβ
- - + + EGF

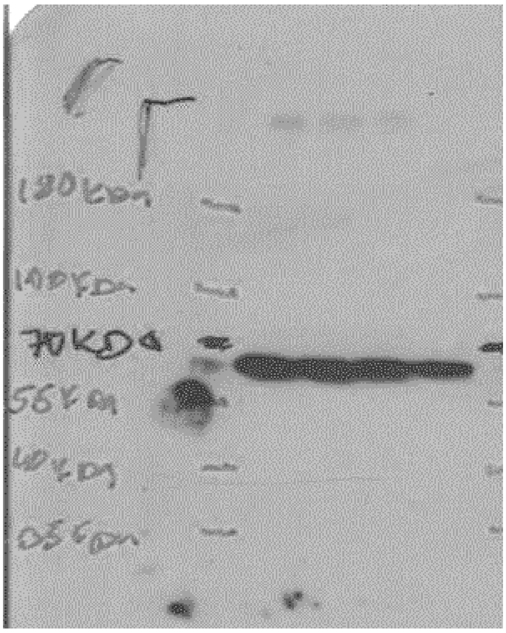

- + - + TGFβ
- - + + EGF

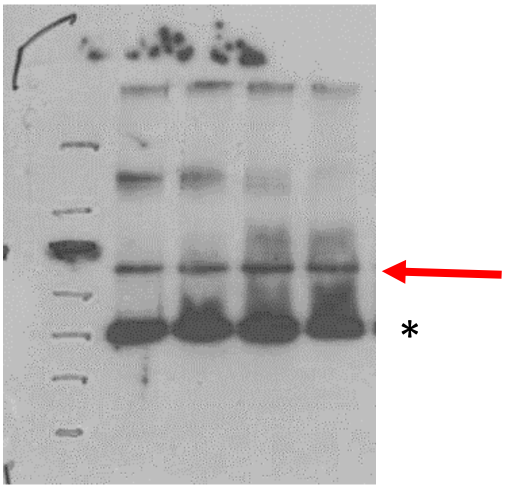

*

- + - + TGFβ
- - + + EGF

* Bands from other antibodies used on the same membrane or unspecific bands

Figure 3A:

β-actin
30 h

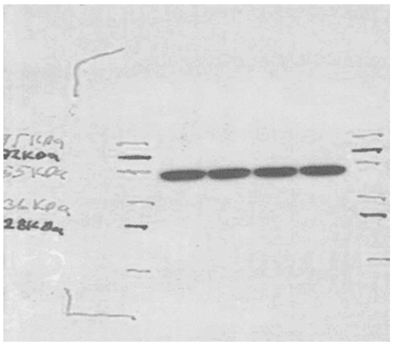

- + - + TGFβ
- - + + EGF

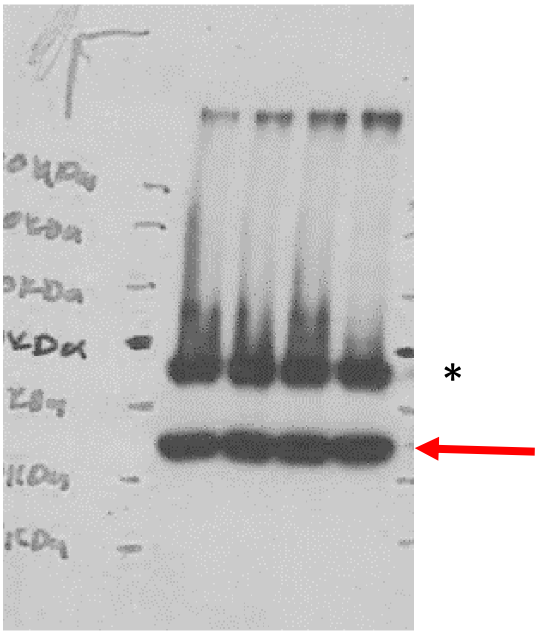

*

- + - + TGFβ
- - + + EGF

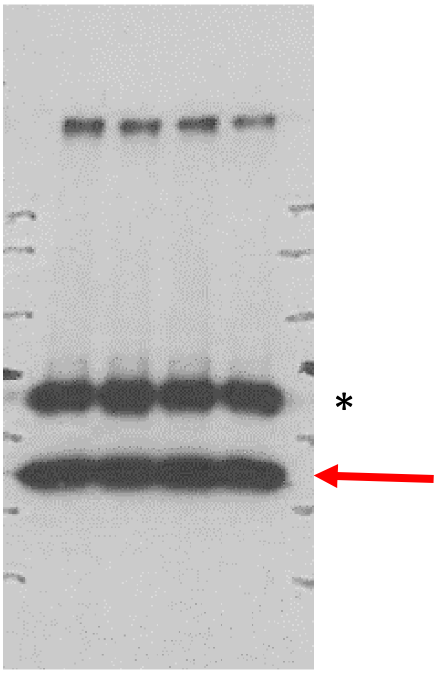

*

- + - + TGFβ
- - + + EGF

* Bands from other antibodies used on the same membrane or unspecific bands

Figure 3B:

pAKT
48 h

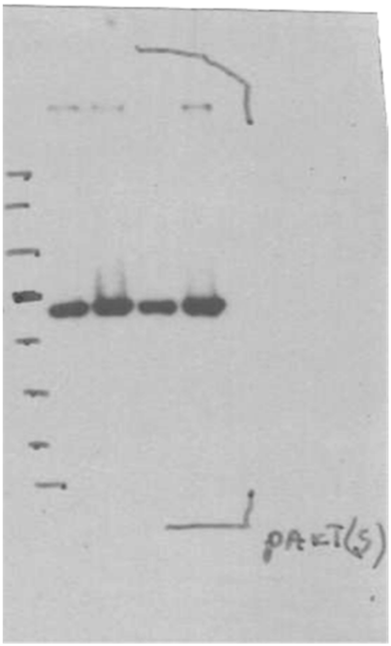

- + - + TGFβ
- - + + EGF

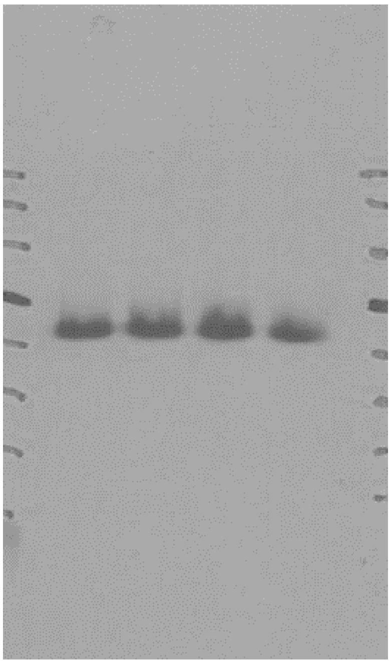

- + - + TGFβ
- - + + EGF

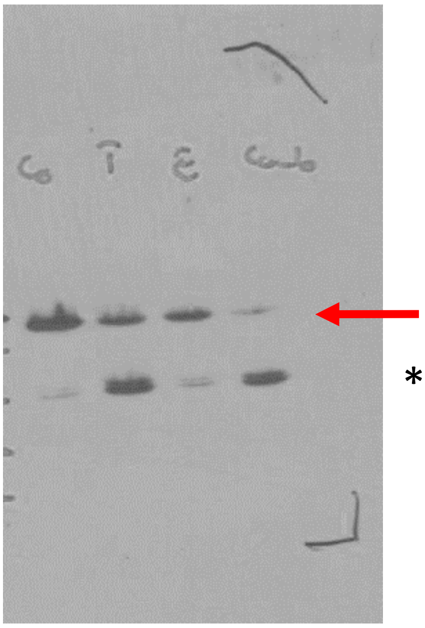

*

- + - + TGFβ
- - + + EGF

* Bands from other antibodies used on the same membrane or unspecific bands

Figure 3B:

AKT
48 h

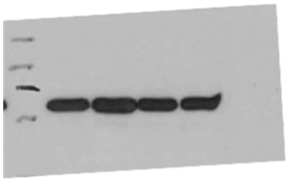

- + - + TGFβ
- - + + EGF

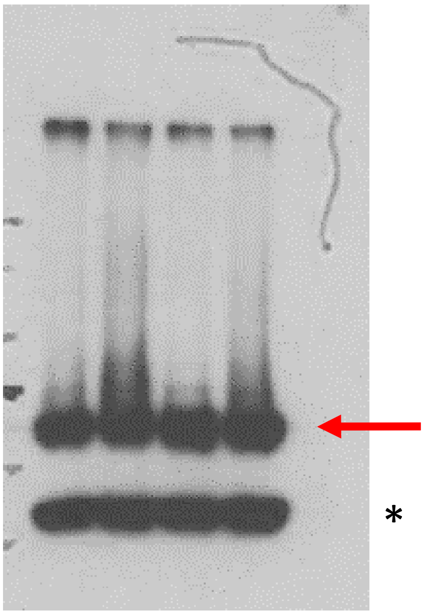

*

- + - + TGFβ
- - + + EGF

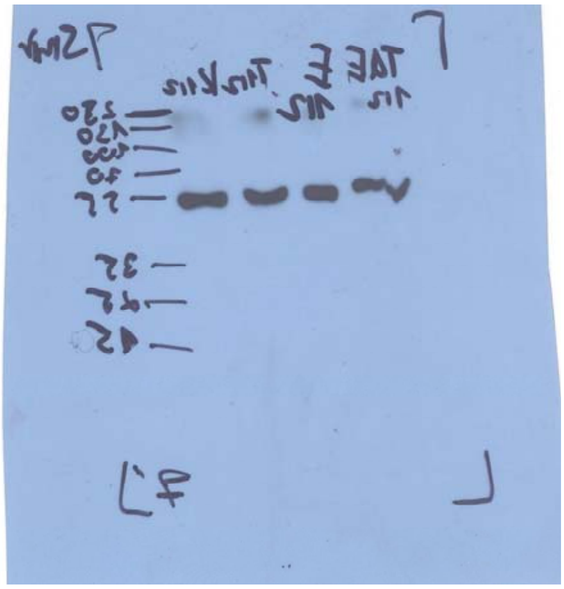

- + - + TGFβ
- - + + EGF

* Bands from other antibodies used on the same membrane or unspecific bands

Figure 3B:

pERK
48 h

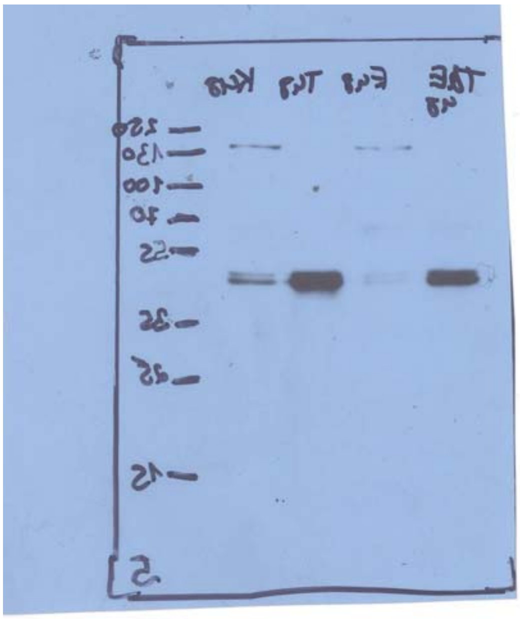

-   + - +   TGFβ

-   - + +   EGF

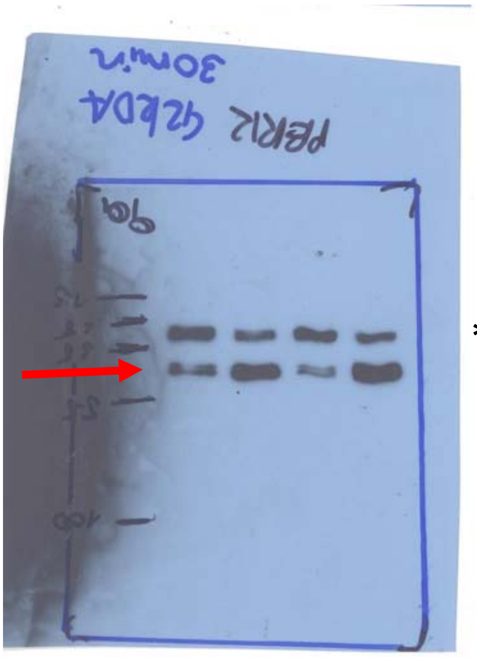

*

-   + - +   TGFβ

-   - + +   EGF

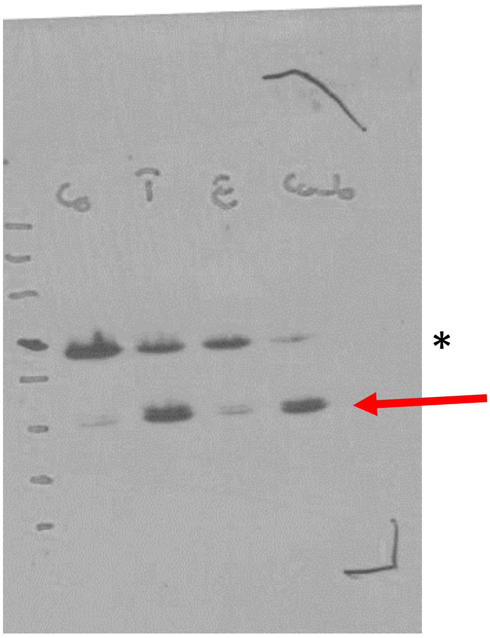

-   + - +   TGFβ

-   - + +   EGF

* Bands from other antibodies used on the same membrane or unspecific bands

Figure 3B:

ERK
48 h

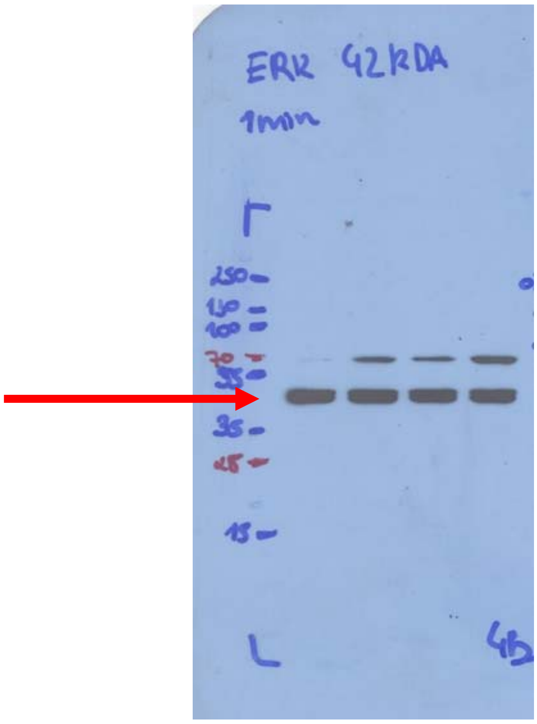

*

- + - + TGFβ

- - + + EGF

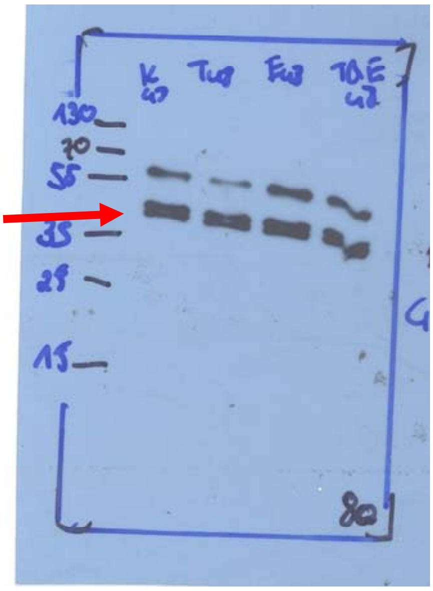

*

- + - + TGFβ

- - + + EGF

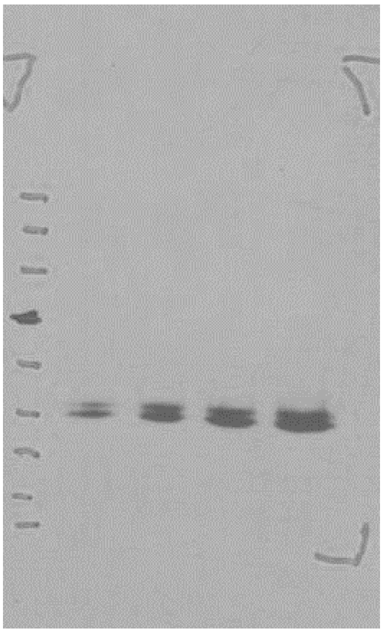

- + - + TGFβ

- - + + EGF

* Bands from other antibodies used on the same membrane or unspecific bands

Figure 3B:

pSMAD2
48 h

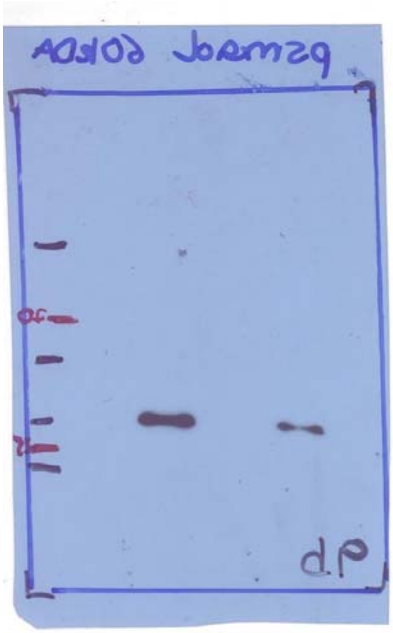

-	+	-	+	TGFβ
-	-	+	+	EGF

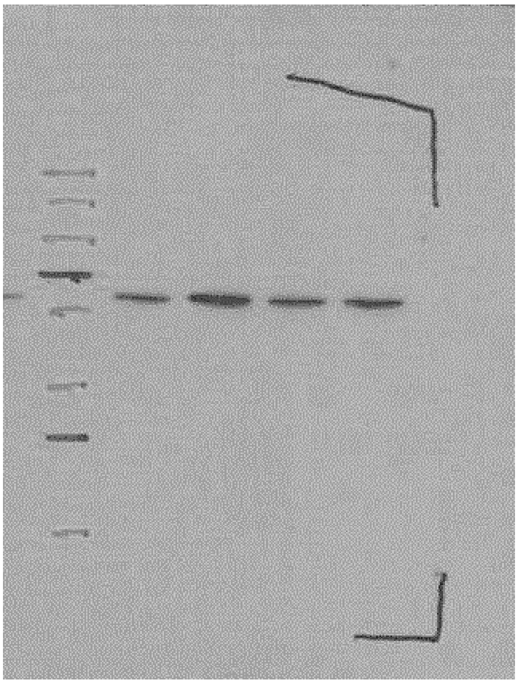

-	+	-	+	TGFβ
-	-	+	+	EGF

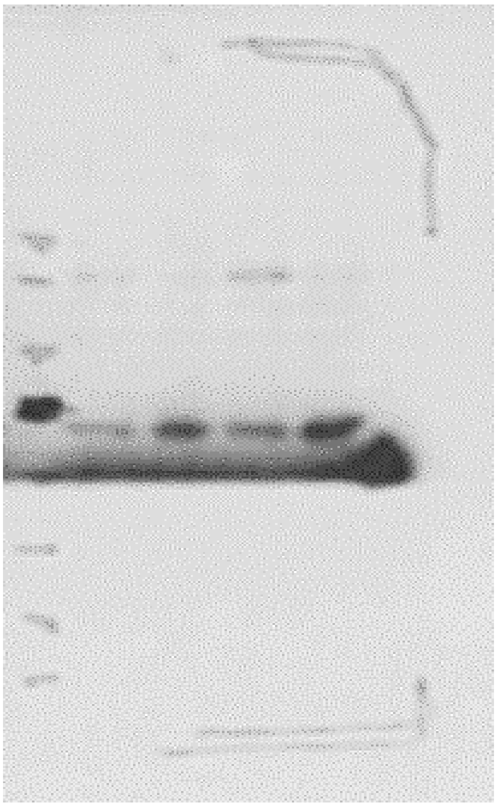

Figure 3B:

SMAD2
48 h

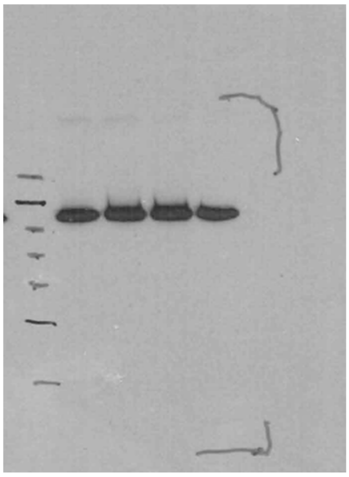

- + - + TGFβ
- - + + EGF

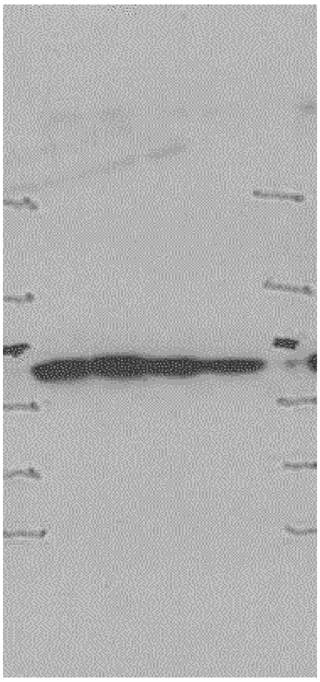

- + - + TGFβ
- - + + EGF

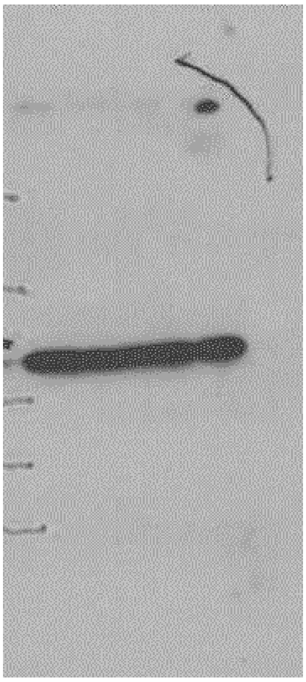

- + - + TGFβ
- - + + EGF

Figure 3B:

β-actin
48 h

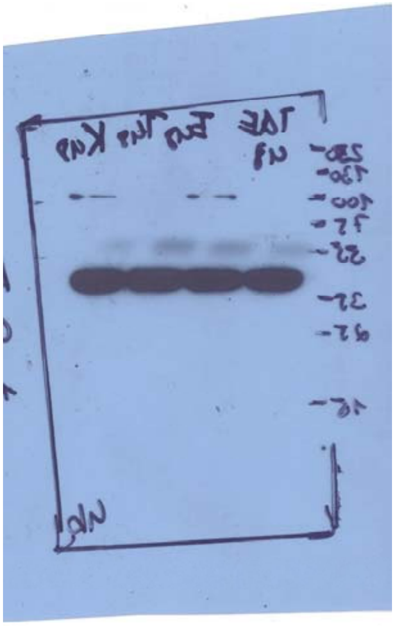

- + - + TGFβ
- - + + EGF

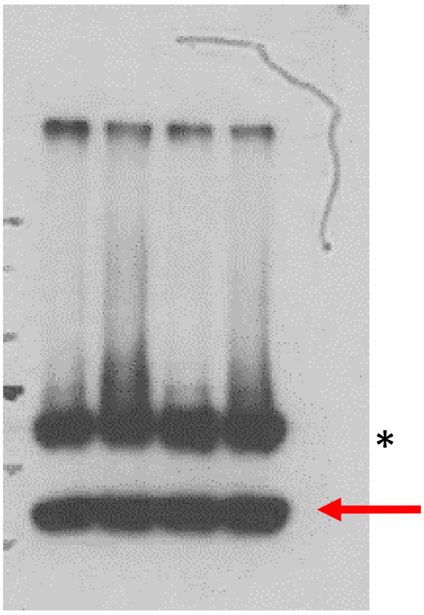

- + - + TGFβ
- - + + EGF

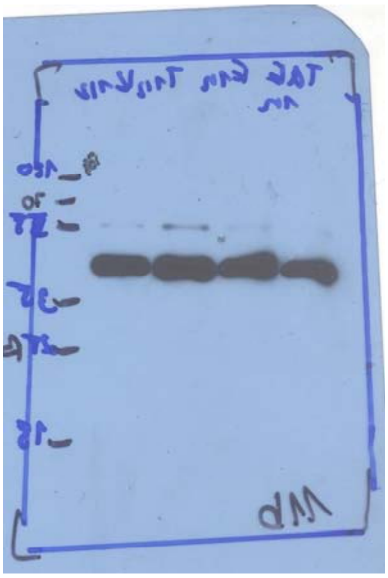

- + - + TGFβ
- - + + EGF

* Bands from other antibodies used on the same membrane or unspecific bands

Supplementary Figure S2:

pERK and ERK
30 min

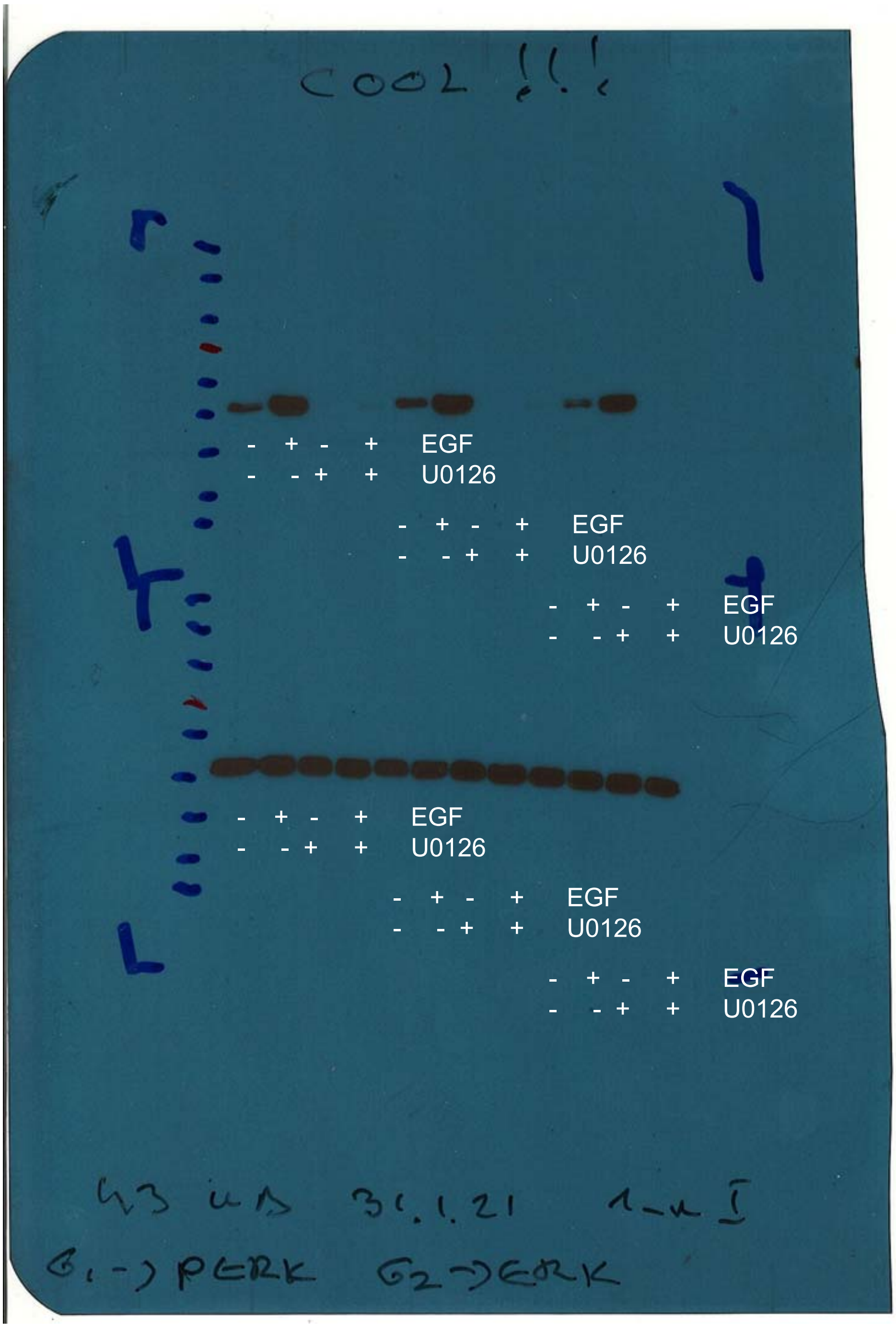

Due to time constraints during the revision, triplicates were seeded from 3 separate flasks, separately treated but run on the same gels

Supplementary Figure S2:

β-actin
30 min

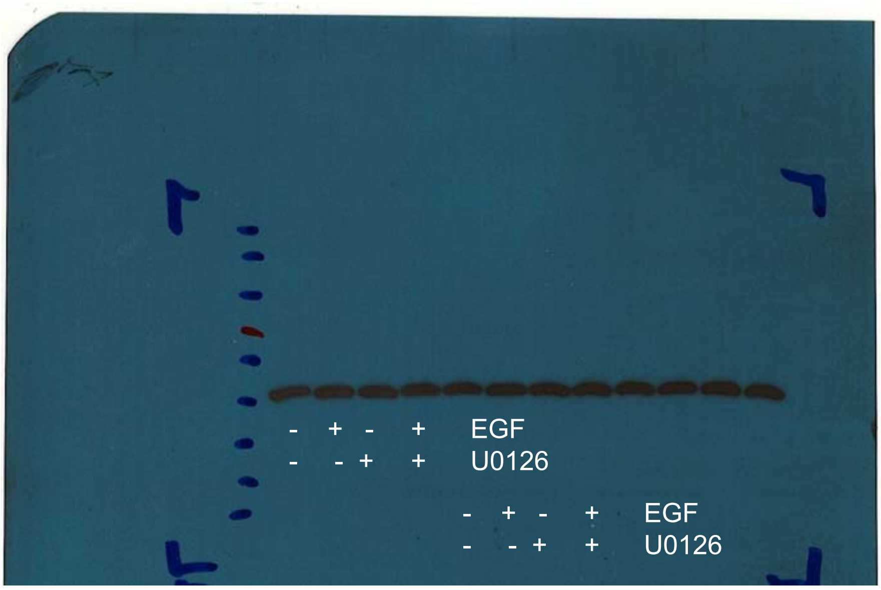

- + - + EGF
- - + + U0126

Due to time constraints during the revision, triplicates were seeded from 3 separate flasks, separately treated but run on the same gels
